# Rationale and design of a multicenter, single-group, open-label trial aiming at investigating the effectiveness of elobixibat for loss of defecation desire in patients with chronic constipation

**DOI:** 10.1016/j.conctc.2022.100958

**Published:** 2022-06-27

**Authors:** Atsushi Yamamoto, Takaomi Kessoku, Kosuke Tanaka, Kota Takahashi, Yuki Kasai, Anna Ozaki, Michihiro Iwaki, Takashi Kobayashi, Tsutomu Yoshihara, Noboru Misawa, Kanji Ohkuma, Akiko Fuyuki, Takuma Higurashi, Kunihiro Hosono, Masato Yoneda, Tomoyuki Iwasaki, Takeo Kurihashi, Machiko Nakatogawa, Ayao Suzuki, Masataka Taguri, Shunsuke Oyamada, Keisuke Ariyoshi, Noritoshi Kobayashi, Yasushi Ichikawa, Atsushi Nakajima

**Affiliations:** a Department of Gastroenterology and Hepatology, Yokohama City University Graduate School of Medicine, Japan; b Department of Gastroenterology, Fujisawa Syounandai Hospital, Japan; c Department of Palliative Medicine, Yokohama City University Hospital, 3-9 Fukuura, Kanazawa-ku, Yokohama, 236-0004, Japan; d Department of Internal Medicine, Iwasaki Naika Clinic, Japan; e Department of Internal Medicine, Kanagawa Dental University Yokohama Clinic, Japan; f Department of Internal Medicine, NamikiKoiso Medical Clinic, Japan; g Department of Data Science, Yokohama City University Graduate School of Medicine, Japan; h Department of Biostatistics, JORTC Data Center, Japan; i Department of Oncology, Yokohama City University Hospital, Japan; j Department of Oncology Yokohama City University Graduate School of Medicine, Japan

**Keywords:** Chronic constipation, Defecation desire, Elobixibat, Bile acid, Loss of defecation desire

## Abstract

**Background:**

Approximately 60% of patients with chronic constipation (CC) have a significantly higher rate of loss of defecation desire (LODD). Bile acids are expected to have a restorative effect on defecation desire (DD) because they lower the rectal sensory threshold, which is an objective index of DD. Elobixibat (EXB) specifically inhibits the ileal bile acid transporter/apical sodium-dependent bile acid transporter, which is a transporter involved in the reabsorption of bile acids in the terminal ileum. This study aims to investigate the LODD improvement rate in patients with CC after 4 weeks of EXB treatment.

**Methods:**

A total of 40 adult patients with CC who meet the eligibility criteria will be enrolled. Patients will receive oral EXB (10 mg/day) for 4 weeks. A patient diary will be provided daily at 4 weeks after treatment. The primary endpoint will be the percentage LODD improvement at week 4 of the treatment period from week 2 of the observation period using questionnaires.

**Ethics and dissemination:**

Ethical approval was obtained from the Yokohama City University Certified Institutional Review Board prior to participant enrolment (approval number: CRB21-008). The results of this study will be submitted for publication in international peer-reviewed journals, and key findings will be presented at international scientific conferences. Participants desiring the results of this study will be directly contacted for data dissemination.

**Trial registration:**

This trial was registered at ClinicalTrials.gov (NCT05165199).

**Protocol version:**

1.0, September 21, 2021.

## 1 Strengths and limitations of this study

• This study is the first exploratory trial to investigate the efficacy of elobixibat for loss of defecation desire in chronic constipation over a 4-week period.
• The primary outcome is the percentage improvement in the loss of defecation desire, whereas the secondary outcome is the fecal bile acid level.
• The study limitations include the small sample size, open-label design, and relatively short treatment period.

## 2 Introduction

Chronic constipation (CC) is a functional disorder frequently encountered in daily clinical practice, with a prevalence of 2%–27% in Japan. In addition, comorbidity with other functional gastrointestinal diseases is common, and decreased quality of life (QOL) has also been reported [[1], [2], [3]]. Establishing a long-term effective treatment for CC is important because of the high frequency of comorbid ischemic heart disease among patients [[4], [5], [6]] and the poor life prognosis of patients with CC, as compared with that of patients without constipation [7]. A web-based questionnaire survey reported that patients with CC exhibited a significantly higher rate of loss of defecation desire (LODD) than healthy adults, with approximately 60% of patients losing their defecation desire (DD) [8]. Patients whose DD improved through treatment expressed greater treatment satisfaction than those whose DD did not improve [8], suggesting that the presence or absence of DD in the treatment environment is related to treatment satisfaction and the importance of DD in the treatment of constipation.

Elobixibat (EXB) is an oral drug for CC that specifically inhibits the ileal bile acid (BA) transporter (IBAT)/apical sodium-dependent BA transporter, a transporter involved in BA reabsorption, in the terminal ileum [9]. EXB was approved for marketing in Japan in January 2018. The IBAT inhibitory action of EXB increases the amount of BAs reaching the colon by inhibiting BA reabsorption, thereby promoting water secretion into the lumen of the large intestine as well as gastrointestinal motility. In addition, BAs are expected to have a restorative effect on DD because they lower the rectal sensory threshold, which is an objective index of DD [10,11]. Nonetheless, in actual clinical practice, the potential restorative effect of EXB, which increases BA in the colon, on DD and duration of recovery has not been investigated. Therefore, this study aims to investigate the efficacy of EXB in patients with CC over a 4-week period.

## 3 Methods

### 3.1 Trial design

**Fig. 1:**
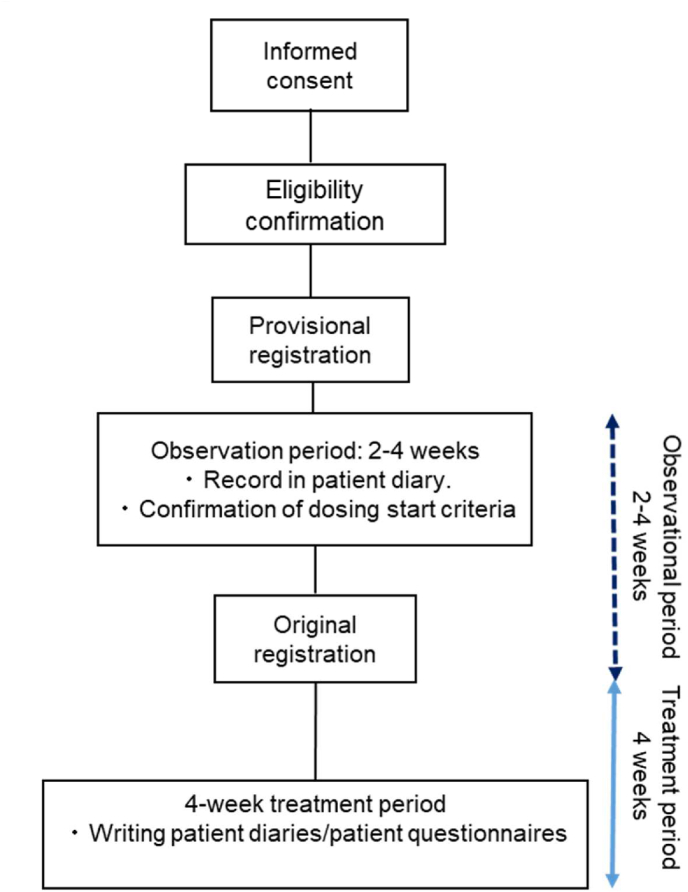
Study design.

The Standard Protocol Items: Recommendations for Interventional Trials (SPIRIT) statement and its checklist were followed in the preparation of this study protocol. Study protocol was shown in Supplementary document 1. This trial was designed as a multicenter, open-label, single-group, investigator-initiated study to investigate the efficacy of 10 mg EXB tablets. Oral treatment will be administered before meals to patients with CC for LODD once daily for 4 weeks (Fig. 1). This clinical study aims to confirm the proof of concept (POC) for EXB therapy. We plan to examine the targets and stages of the subsequent phase. Patient questionnaires will be administered daily, and fecal samples will be collected at baseline and at 4 weeks after the intervention. The study plan involves recruiting 40 adult patients with CC and LODD from five institutions, namely, Yokohama City University Hospital, Fujisawa Syounandai Hospital, Iwasaki Naika Clinic, Kanagawa Dental University Yokohama Clinic, and NamikiKoiso Medical Clinic. This trial was registered at ClinicalTrials.gov (NCT05165199) on December 4, 2021. The trial results will be reported in accordance with the Transparent Reporting of Evaluations with Nonrandomized Designs (TREND) statement [12].

### 3.2 Rationale for treatment dose, mode, duration, and endpoint

The European Medicines Agency (EMA) guidelines state that QOL assessment is important; furthermore, a web-based questionnaire survey in 2020 reported that patients with CC exhibited a higher rate of LODD than healthy adults and that patients whose DD improved with treatment had a greater level of treatment satisfaction [8]. We hypothesize that EXB increases the amount of BAs that enter the lumen of the large intestine and that these BAs lower the rectal sensory threshold, thereby leading to the realization of DD. In this study, the primary endpoint is “confirmation of recovery of LODD,” which is considered to be related to treatment satisfaction. In the literature [13], an improvement in the number of spontaneous bowel movement, among others, was observed after 2 weeks of treatment. Because EXB is effective in improving constipation at an early stage, we believe that early recovery of LODD can be expected in actual clinical practice. In this study, we set “confirmation of recovery of LODD” at the 4-week administration, which is the usual interval between visits.

### 3.3 Drug supply

This clinical trial will be open, and the patient registration center, doctors, and patients will be informed of the allocation results. The manufactured EXB hydrate tablets (5 mg) are prescribed by the physician and provided by the patient registration center (personally dispensed by the pharmacy manager).

### 3.4 Sample-size estimation

No studies have investigated the recovery of LODD by treating CC via EXB administration. According to a web-based survey [8] of 20,986 Japanese patients conducted at Yokohama City University, the rate of pre-treatment LODD (no DD: none + almost none) for Rome IV CC and in healthy adults was 57.4% (no DD: none + almost none; 1,484/2,587 cases) and 8.3% (214/2,587 cases), and the rate of DD disappearance in outpatient treatment cases was 27.1% (120/443 cases). Because the subjects in this study were “patients without DD” who had already received CC treatment within the scope of usual care, the expected LODD improvement rate was estimated at 45%, which was lower than 62.1%. The threshold rate of spontaneous change in DD was estimated at 20%. In cases in which the threshold was 20%, the expected value was 45% (one-sided α: 0.025; total number of cases: 37; power: 91.6%), which is statistically significant when the number of cases is ≥ 13 out of 37. Although, within the scope of normal practice, we considered some patients to have possibly withdrawn from the study after provisional enrollment, we considered 40 cases to be an appropriate target number of patients for this study. The number of patients with CC at each institution who were expected to meet the eligibility criteria, including “no DD,” was examined, and it was concluded that the number of patients could be accumulated within the study period without challenges (the planned enrollment period was approximately 6 months).

### 3.5 Eligibility

Following the acquisition of informed consent, eligible cases are assigned a registration number and recorded in the screening list; their eligibility is determined according to the inclusion and exclusion criteria (Table 1). If no eligibility issues are identified, the investigator/sub-investigator and investigative staff will enter the necessary data into the electronic data capture system for enrolment. Enrolment will subsequently be completed by assigning the patient enrolment number.

**Table 1:** Patient inclusion and exclusion criteria.

	Inclusion criteria	Exclusion criteria
At the time of temporary registration		
1	Patients diagnosed with CC according to the Rome IV criteria for the diagnosis of CC	Patients with organic constipation or suspected of having organic constipation
2	Age: ≥20 years (at the time of consent acquisition)	Patients with or suspected of having functional ileus
3	Sex: Any	Patients with or suspected of having inguinal hernia
4	Outpatients	Patients with a history of open abdominal surgery within 12 weeks prior to consent acquisition (excluding appendicitis resection)
5	Patients from whom written consent can be obtained	Patients with a history of surgical or endoscopic procedures related to gallbladder resection and papillotomy
6	Patients who can report defecation, etc., in the patient diary	Patients with complications of malignancy; however, patients who had undergone radical surgery or completed chemotherapy or radiotherapy may be registered
7		Pregnant women, lactating women, women who may be currently pregnant, or women who use contraception while participating in the study; patients who do not provide their consent for participation
8		Patients with serious renal, hepatic, or cardiac disease
9		Patients with a history of drug allergy to the study drug
		Patients participating in other clinical research or who participated in other clinical studies within 4 weeks prior to the provision of consent; patients who participated in the study but not in observational studies
		Any other information that the principal investigator or sub-investigator deems inappropriate for participation in this research (e.g., patient disability)
At the time of registration: Dose-initiation criteria		
1	Patients with LODD* at week 2 of the observation period (1 week before treatment initiation)* “LODD” refers to patients whose “presence or absence of DD” on the patient questionnaire was “4: almost never” or “5: never”	Patients whose doses of concomitantly restricted medications were increased during the observation period
2		Patients who used concomitantly prohibited drugs during the observation period

CC: chronic constipation, LODD: loss of defecation desire.

### 3.6 Randomization and masking

Not applicable.

### 3.7 Keycode break

Not applicable.

### 3.8 Harm and adverse event monitoring

Adverse events (AEs) are any unwanted or unintended side effects, including abnormal laboratory test values or abnormal vital signs; symptoms; or illnesses that develop during the trial. The causal relationship with the investigational drug will be considered irrelevant. The principal or sub-investigator will assess the severity of AEs. Any AE that meets any of the following criteria will be considered a severe AE (SAE): death, life threat, hospitalization requirement or prolonged hospitalization for treatment, disability, disability threat, congenital disease, anomaly in offspring, or other serious conditions. If an SAE occurs, the principal or sub-investigator will treat the SAE as appropriate and immediately report the details to the hospital director, the Yokohama City University Certified Institutional Review Board, and the study drug supplier. If a serious health hazard arises due to participation in this study, coverage benefits can be received from insurance carried by a principal investigator, provided, however, that compensation may be reduced or not compensated if it is proven that the health hazard was caused by the research subject's own wilful act or gross negligence. In addition, if there is no causal relationship between the newly occurring health hazard and the deterioration of the originally affected disease, it is not covered by compensation. After completion of the study by each research subject, the investigators will make efforts to provide the best medical care obtained from the results of the research.

### 3.9 Study procedures

The investigator or sub-investigator will perform all observations, tests, investigations, and evaluations according to the descriptions summarized in Table 2. In the patient demographic, we plan to collect information on comorbidities and concomitant medications. After treatment initiation, drug returns and blood test results will be evaluated to monitor adherence at each visit. Stool samples will be collected and stored to assess BA levels.

**Table 2:** Schedule for observations, tests, and assessments.

	Informed consent	Observation period		Treatment period
		V1	V2	V3/EOT
Study week		Provisional registration	Final registration	Week 4
Visit window		–	2–4 weeks after registration	±7 days
Informed consent	○			
Inclusion/exclusion criteria		○	○	
Demographics		○		
Vital signs			○	○
Subjective/objective findings			○	○
Blood tests			○	○
Biochemical test			○	○
Provisional registration		○		
Enrollment/initiation criteria			◎	
Blood and stool collection for exploratory research			●	●
Prescription of the study drug			○	
Checking the medication status				○
Review of concomitant medications		○	○	○
Review of adverse events		○	○	○
Patient diary confirmation			○	○
Patient questionnaire confirmation			○	○
JPAC-QOL, CSS survey			○	○

JPAC-QOL, Japanese version of Patient Assessment of Constipation Quality Of Life; CSS, constipation severity score.

### 3.10 Concomitant treatment

*Restricted concomitant drugs and therapies:* The administration of the following medications and therapies is prohibited from the start of the observation period to the end of the treatment period: CC medications (including over-the-counter medications and supplements) and therapies used prior to enrollment should not be changed in principle until the end of the treatment period. However, dose reduction or discontinuation will be acceptable. The use of enemas, suppositories, and stool extraction is permitted only when adequate defecation is not observed. Prohibited concomitant medications from the observation period to the end of the treatment period include BA transporter inhibitors other than the study drug, BA preparations (ursodeoxycholic acid, chenodeoxycholic acid, and dehydrocholic acid), antacids containing aluminum (sucralfate hydrate, aldioxa, etc.), cholestyramine, and cholestymid. *Concomitant medications:* The following drugs should be used with caution during treatment (until the final dose): digoxin, dabigatran, etexilate methanesulfonate, and midazolam.

### 3.11 Criteria and procedure for withdrawal from the study

The principal investigator or sub-investigator will terminate the participation of a patient enrolled in the clinical trial if any of the following applies: (1) withdrawal from the clinical trial is requested by the patient; (2) it is found after registration that the patient does not meet the inclusion criteria or conforms to one or more exclusion criteria; (3) drugs or therapies whose concomitant use is prohibited are being administered; (4) it is difficult to continue the clinical trial owing to the occurrence of AEs or for other reasons; and (5) continuation of the clinical trial is considered inappropriate by the principal investigator or others.

### 3.12 Study endpoints

The study endpoints are listed in Table 3.

**Table 3:** Study endpoints.

Primary endpoint	Secondary endpoints	
<Efficacy endpoint>	<Efficacy endpoint>	<Safety endpoint>
Percentage improvement in LODD at week 4 of the treatment period from week 2 of the observation period	(1) Changes in the following items in each week of the treatment period and comparison of week 4 of the treatment period with week 2 of the observation period: 1. Presence of DD: patient questionnaire 2. Satisfaction with DD: a patient questionnaire 3. Satisfaction with straining: patient questionnaire 4. Degree of straining: patient diary 5. Presence of a sense of incomplete evacuation: patient diary 6. Satisfaction with treatment: patient questionnaire 7. SBM frequency 8. CSBM frequency 9. Stool hardness based on the BSFS	Incidence rate of diseases
	(2) Comparison of the following items at week 4 of the treatment period with week 2 of the observation period: 1. Constipation score: CSS 2. JPAC-QOL score 3. Absolute value and percent composition of bile acids in feces	
	(3) Changes in the time from taking EXB to defecation in each week during the treatment period	
	(4) Consideration of the relationship between the evaluation items	

BSFS, Bristol Stool Form Scale; CSBM, complete spontaneous bowel movement; CSS, constipation severity score; DD, defecation desire; EXB, elobixibat; JPAC-QOL, Japanese version of the Patient Assessment of Constipation Quality of Life; LODD, loss of defecation desire; SBM, spontaneous bowel movement.

### 3.13 Analysis population

The set of patients to be analyzed will be determined before logging the data of each patient and defined as follows: the modified intention-to-treat—that is, the full analysis set (FAS) and per-protocol set (PPS)—will be used to assess primary efficacy. The FAS will include all patients except for those who met any of the following criteria: (1) cases of serious clinical trial protocol violations (violations of consent acquisition, serious violations of clinical trial procedures, etc.); (2) cases in which the study drug had never been administered; and (3) cases in which no efficacy-related endpoints were measured. The PPS will be a subpopulation of the FAS, excluding cases with clinical trial protocol violations, such as *ex post facto* findings of violations of the inclusion criteria or the use of drugs or treatments whose concomitant use is prohibited. The safety analysis set (SAS) will be used for safety assessment and will include all cases in which the investigational drug was administered at least once.

### 3.14 Statistical analysis

The main analysis will be conducted on the FAS, and the exact test for the binomial proportion will be conducted for the percentage improvement in DD from week 2 of the observation period to week 4 of the treatment period (the percentage of subjects who fall into categories other than “not at all” or “hardly at all”), assuming that the threshold (mother proportion in the null hypothesis) is 20%. A one-tailed (upper-tailed) test will be conducted, with a significance level of 2.5% on each side. Point estimates for the percentage improvement in LODD and exact two-sided 95% confidence intervals for the binomial proportions will be calculated. Discontinued cases will contribute solely to the denominator in the main analysis (missing data in the numerator will not be supplemented). In addition, as part of the sensitivity analysis, missing data for discontinued cases will be supplemented according to the criteria in “Primary endpoint” if you have been taking study drugs for more than a week. However, the completion of missing data in the sensitivity analysis will only be performed when completion by evaluation at the time of dose discontinuation is considered appropriate, based on a summary of the missing status of the primary endpoint.

The presence or absence of DD will be assessed using a 5-point scale in the patient questionnaire: 1, always; 2, almost always; 3, a little; 4, almost never; and 5, never. In this study, DD frequency was measured using a 5-point scale, and subjects with no DD were enrolled. An analysis similar to the primary analysis for PPS will be performed as a secondary analysis of the primary endpoint. A subgroup analysis will be performed in which the subject background factors will be grouped into appropriate categories.

### 3.15 Interim analysis

Not applicable.

### 3.16 Data management, central monitoring, and audit

The sites where the investigators will perform the trial will maintain the individual records of each patient as source data, which will include a copy of the informed consent form, medical records, laboratory data, and other records or notes. All data will be collected by an independent data management center. The data management center will oversee the interstudy data-sharing process. Clinical data entry, data management, and central monitoring will be performed using electronic data capture software, VIEDOC 4 (PCG Solutions, Stockholm, Sweden). No auditing will be planned.

### 3.17 Study flow and schedule of enrolment, interventions, and assessments

A flowchart of this study is shown in Fig. 1, and the study schedules are presented in Table 3.

### 3.18 Protocol amendments

When protocol is modified, the modification is reported to Yokohama City University of Medicine Clinical Research Review Board immediately. After protocol modifications is approved, the modification is reported to jRCT.

### 3.19 Patient and public involvement

In this trial, patients will be involved in the recruitment and conduct of the study. In particular, the development of the research question and outcome measures will be based on the priorities, experiences, and preferences of patients. The results of this study will be disseminated during examination to participants who are interested in the results. The intervention burden will be assessed by patients prior to the commencement of the trial; patient satisfaction with the treatment will be assessed as part of the post-intervention assessment.

### 3.20 Ethics and dissemination

The study protocol complies with the principles of the Declaration of Helsinki and Clinical Trial Act published by the Ministry of Health, Labour and Welfare, Japan. We obtained approval for this study from the Yokohama City University Certified Institutional Review Board on October 7, 2021. The protocol and informed consent form were approved by the Yokohama City University Certified Institutional Review Board. Written informed consent for participation in the study is obtained from all participating patients. The results of this study will be disseminated face-to-face to participants who indicate interest in obtaining the results. The results of this study will be submitted for publication in international peer-reviewed journals, and key findings will be presented at international scientific conferences.

## 4 Discussion

This is the first clinical study to demonstrate the use of EXB for treating LODD in patients with CC. In general, CC treatment aims to improve the frequency of bowel movement, defecation-difficulty symptoms (a sense of incomplete evacuation and straining), and abdominal symptoms. Therefore, the majority of endpoints in previous randomized controlled trials investigating various laxatives for CC have focused on the frequency of bowel movement and QOL [[13], [14], [15], [16]]. Nonetheless, in our previous Internet survey, patients with CC whose bowel movement had disappeared before treatment initiation improved their loss of bowel movement with constipation treatment. Patients whose bowel movement improved with constipation treatment were significantly more satisfied with the treatment than those whose bowel movement did not improve [8]. Constipation in patients with stroke was also associated with higher rectal sensory thresholds, bowel thresholds, and thresholds of urgency for bowel movements, which correlated with PAC-QOL [17]. Symptoms associated with decreased rectal sensitivity have been reported to include decreased defecation frequency, increased pain during defecation, long-term toilet use, increased use of disimpaction/enema, and hard stool [18].Therefore, it is important to improve patient satisfaction and defecation-specific QOL by restoring DD when treating patients with CC who have lost their DD.

The only reports on drug therapy for DD are those in which Daikenchuto was used in pediatric patients with severe constipation, and the sensory threshold was lowered [19]. However, a randomized controlled trial using Daikenchuto for functional constipation in adult women revealed no significant difference in the sensory threshold of constipation, as compared with placebo [20]; this phenomenon is yet to be fully clarified. A placebo-controlled, double-blind, randomized crossover study on lubiprostone use in patients with irritable bowel syndrome with constipation also revealed no significant difference in the sensory threshold of stool intention [21]. Therefore, the establishment of drug therapy for constipation treatment that focuses on the recovery of DD is important for improving patient satisfaction.

BAs are often reported to lower the rectal sensory threshold and promote proximal colonic motor responses [10,11,22]. Based on these previously reported results, we believe that a BA increase is the key to LODD resolution in patients with CC. Therefore, we devised a POC trial using EXB, which has a BA-increasing effect in the large intestine [23].

Our study protocol possesses the following strengths: (1) the assessment of LODD recovery as the primary endpoint, (2) measurements of Bristol Stool Form Scale score and (3) bowel movement related to defecation-specific QOL as secondary endpoints, and (4) measurement of fecal BA. Nevertheless, our study also has the following limitations: lack of comparisons with (1) other laxatives and (2) placebo.

### 4.1 Conclusions

This is the first study for the assessment of the efficacy of EXB in recovering the LODD in constipation patients with DD.

## Ethics approval

The Yokohama City University Certified Institutional Review Board approved the study protocol on October 7, 2021 (approval number: CRB21-008). This trial was registered at ClinicalTrials.gov (NCT05165199) and Japan Registry of Clinical Trials (jRCTs031210477).

## Confidentiality

The data will be retained in accordance with the Clinical Trial Act. Each participant will be registered using a unique identification (ID) number at entry. The master list linking the participants’ personal information and their ID numbers will be maintained in a separate locked cabinet on a password-protected hard drive. Data will be analyzed solely by ID numbers. Records will be retained for 5 years after study completion and subsequently destroyed by the data management center.

## Funding

This work was supported by 10.13039/100014421EA Pharma and Mochida Pharma (Tokyo, Japan). The funder had no role in the study design, data collection, data analysis, data interpretation, or writing of the report. The corresponding author had full access to all data and had the final responsibility regarding the decision to submit for publication.

## Authors’ contributions

AY, T. Kessoku, and AN conceived the study. AY and T. Kessoku conducted the feasibility phase work. Participant recruitment and follow-up will be performed by KT, KT, YK, AO, MI, T. Kobayashi, TY, NM, KO, AF, TH, KH, MY, TI, TK, MN, and AS. Data management will be performed by KA. MT and SO performed bioinformatic analysis. Data analysis and interpretation will be conducted by MT, SO, NK, YI, and AN. All authors have read and approved the final manuscript.

## Data sharing statement

Data are available upon reasonable request.

## Declaration of competing interest

ANa reports grants and research support from Gilead, Mylan EPD, EA Pharma, Kowa, Taisho, and Biofermin. ANa is a consulting adviser for Gilead, Boehringer Ingelheim, BMS, Kowa, Astellas, EA Pharma, and Mylan EPD. The other authors declare no conflicts of interest.

## Data Availability

Data will be made available on request.
