# Supplementary material for: Rationale and design of a multicenter, single-group, open-label trial aiming at investigating the effectiveness of elobixibat for loss of defecation desire in patients with chronic constipation
Source: Contemp Clin Trials Commun. 2022 Jun 27;28:100958. doi: 10.1016/j.conctc.2022.100958 (PMC9260435; doi:10.1016/j.conctc.2022.100958)
Supplement: Multimedia component 1 [file mmc1.docx]

Efficacy of elobixibat for recovery of loss of

defecation desire in patients with chronic constipation. Multicenter, single-group, open label study.
(№ R-LODD study)

principal investigator

Yokohama City University Hospital

Department of Palliative Medicine KESSOKU Takaomi

Ver. 1.0

Created On: September 21, 2021

creation-time

| version number | creation date |
| --- | --- |
| Ver. 1.0 | Sep. 21, 2021 |
|  |  |
|  |  |
|  |  |

Confidentiality Agreement

This research protocol is confidential and will be provided to the principal investigator, principal investigator, subordinate investigators, research collaborators, accredited clinical research review committee, EA Pharma, Inc., and Mochida Pharmaceutical Co.

This research protocol may not be disclosed to any third party or used for any purpose other than the purpose of this research without the written consent of the principal investigator, except when explaining the contents of this research to subjects.

**Table of contents**

[**0. An Overview** 7](#_Toc80203950)

[**1. Purpose of this study** 10](#_Toc80203951)

[**2. Background and the scientific rationale for the study design** 10](#_Toc80203952)

[2.1. Target disease 10](#_Toc80203953)

[2.2. Standard Treatment 11](#_Toc80203954)

[2.3. Treatment 11](#_Toc80203955)

[2.4. Study design and primary endpoints 12](#_Toc80203956)

[2.5. Significance of this study 12](#_Toc80203957)

[**3. Study Drug Information** 12](#_Toc80203958)

[3.1. Study drug 12](#_Toc80203959)

[3.2. Management of the study drugs 13](#_Toc80203960)

[3.3. Ensuring the quality of the study drugs 13](#_Toc80203961)

[**4. Criteria and definitions used in this study** 13](#_Toc80203962)

[**5. Patient selection policy** 15](#_Toc80203963)

[5.1. Selection criteria 15](#_Toc80203964)

[5.2. Exclusion criteria 15](#_Toc80203965)

[**6. Research plan** 16](#_Toc80203966)

[6.1. Study design 16](#_Toc80203967)

[6.2. Target sample size 16](#_Toc80203968)

[6.3. Study period 16](#_Toc80203969)

[6.4. Institutional registration and case registration 17](#_Toc80203970)

[6.5. Treatment planning 19](#_Toc80203971)

[**7. Observation, examination, investigation, and evaluation items** 22](#_Toc80203972)

[7.1. Schedule table 22](#_Toc80203973)

[7.2. Implementation schedule and evaluation items 23](#_Toc80203974)

[7.3 Evaluation method 24](#_Toc80203975)

[**8. Method of acquiring consent** 24](#_Toc80203976)

[8.1. Informed consent 24](#_Toc80203977)

[8.2. Response to consultations by subjects and their associated persons 25](#_Toc80203978)

[8.3. When informed consent is obtained from the representative, etc. 25](#_Toc80203979)

[8.4. When obtaining informed assent 25](#_Toc80203980)

[8.5. When obtaining informed consent for test subjects is not necessary 25](#_Toc80203981)

[**9. Evaluation items** 25](#_Toc80203982)

[9.1. Primary endpoint 25](#_Toc80203983)

[9.2. Secondary endpoints 26](#_Toc80203984)

[9.3. Safety endpoint 26](#_Toc80203985)

[**10.** **Discovery research** 26](#_Toc80203986)

[10.1. Timing of sample collection and transport 26](#_Toc80203987)

[10.2. Control, storage, and disposal of samples 26](#_Toc80203988)

[10.3. Withdrawal of consent for the use of the sample 27](#_Toc80203989)

[**11. Storage of samples and information** 27](#_Toc80203990)

[11.1. Storage and disposal of samples 27](#_Toc80203991)

[11.2. Storage period for information 27](#_Toc80203992)

[11.3. Secondary use of samples and information 28](#_Toc80203993)

[11.4. Use of samples and information as biobanks 28](#_Toc80203994)

[**12. Handling of diseases** 28](#_Toc80203995)

[12.1. Definition of disease 28](#_Toc80203996)

[12.2. Evaluation of diseases 28](#_Toc80203997)

[12.3. Causal relationships with research. 29](#_Toc80203998)

[12.4. Causal relationship with research drugs 29](#_Toc80203999)

[12.5. Predictability 29](#_Toc80204000)

[12.6. Measures to be taken in the event of an outbreak of a disease. 30](#_Toc80204001)

[**13. Discontinuation criteria and procedures** 32](#_Toc80204002)

[13.1 Discontinuation criteria 32](#_Toc80204003)

[13.2 Discontinuation procedure 33](#_Toc80204004)

[13.3 Post-treatment after completion (discontinuation) of protocol treatment 33](#_Toc80204005)

[**14. Discontinuation of the study** 33](#_Toc80204006)

[**15. Efficacy and safety evaluation committee** 34](#_Toc80204030)

[**16. Statistical analysis** 34](#_Toc80204031)

[16.1. Analysis set 34](#_Toc80204032)

[16.2. Rationale for setting the target number of patients 34](#_Toc80204033)

[16.3. Statistical analysis 35](#_Toc80204034)

[16.4. Interim analysis 37](#_Toc80204035)

[16.5. Procedures for handling missing, non-adopted, and abnormal data 37](#_Toc80204036)

[16.6. Procedures for changing the statistical analysis plan 37](#_Toc80204037)

[**17. Quality control and assurance** 38](#_Toc80204038)

[17.1. Source documents and the inspection thereof 38](#_Toc80204039)

[17.2. Data management 38](#_Toc80204040)

[17.3. Monitoring 38](#_Toc80204041)

[17.4. Audit 39](#_Toc80204042)

[**18. Ethical matters** 39](#_Toc80204043)

[18.1 Rules to be observed 39](#_Toc80204044)

[18.2. Handling of personal information, etc. 39](#_Toc80204045)

[18.3. Expected benefits and disadvantages to subjects in accordance with research participation, etc. 39](#_Toc80204046)

[18.4. Handling of study results (including incidental findings) for subjects 40](#_Toc80204047)

[**19．Cost burden and reward for examinees** 40](#_Toc80204048)

[**20．Compensation for health damage** 40](#_Toc80204049)

[**21. Conflict of interest management related to study, including funding sources for research** 40](#_Toc80204050)

[21.1. Funding resources and financial relationships 40](#_Toc80204051)

[21.2. Conflicts of interest management 40](#_Toc80204052)

[**22.** **Control of compliance, changes, and non-compliance with the study protocol (deviations from the study protocol, etc.)** 41](#_Toc80204053)

[22.1. Compliance with the study protocol 41](#_Toc80204054)

[22.2. Changes to the study protocol 41](#_Toc80204055)

[22.3. Control of noncompliance (deviations from the study protocol, etc.) 41](#_Toc80204056)

[**23. Periodic reports** 42](#_Toc80204057)

[23.1. Periodic reports to the accredited clinical research review board 42](#_Toc80204058)

[23.2. Periodic report to the Minister of Health, Labor, and Welfare 42](#_Toc80204059)

[**24. Disclosure of study information and publication of results** 42](#_Toc80204060)

[24.1. Enrollment of studies 42](#_Toc80204061)

[24.2. Publication of research results 43](#_Toc80204062)

[24.3. Promulgation via academic societies, etc. 43](#_Toc80204063)

[**25．Attribution of study outcomes (Intellectual Property Rights)** 43](#_Toc80204064)

[**26. System for conducting research** 43](#_Toc80204065)

[**27.** **articipating centers and institutional investigators** 43](#_Toc80204066)

[**28. References** 43](#_Toc80204067)

[**29. Appendix** 44](#_Toc80204068)

**abbreviated table**

| acronym | formal name |
| --- | --- |
| ALP | Alkaline phosphatase |
| ALT | Alanine aminotransferase |
| AST | Aspartate aminotransferase |
| BS | Bristol Stool |
| CSBM | Complete Spontaneous Bowel Movements |
| CK | Creatinekinase |
| Cl | chlorine |
| CSS | Constipation Scoring System |
| EOT | End of treatment |
| EDC | Electronic data capture |
| EMA | European Medicines Agency |
| eCRF | electronic case report form |
| FAS | full analysis set |
| jRCT | Japan Registry of Clinical Trials |
| gamma-GTP | Gamma-glutamyl transpeptidase |
| HDL | High density lipoprotein |
| IBAT/ASBT | Ileal bile acid transporter/ apical sodium-dependent bile acid transporter |
| PAC-QOL | Patient assessment of constipation quality of life |
| k | potassium |
| LDL | Low density lipoprotein |
| Na | sodium |
| PPS | per protocol set |
| quality of life | Quality of life |
| Rome IV | -. |
| SBM | Spontaneous Bowel Movements |
| SAS | safety analysis set |

# **0. Overview**

| Study title | Efficacy of elobixibat for recovery of loss of defecation desire in patients with chronic constipation. Multicenter, single-group, open label study. |
| --- | --- |
| Name of study drug | Elobixibat 5mg |
| Target disease | chronic constipation |
| Research Methods, Research Design | A multicenter, single-arm, open-label, before-and-after study |
| research period | Registration period: from the date of jRCT publication (date of notification by the Minister of Health, Labour and Welfare) to July 31, 2022  Study period: From the date of publication of the jRCT (date of notification to the Minister of Health, Labour and Welfare) to March 31, 2023 (approximately 2 years)  Planned duration of subject participation: up to 9 weeks from obtaining consent  Observation period: 2~4 weeks  Treatment period: 4 weeks (maximum 5 weeks) |
| Research implementation plan № | R-LODD study |
| purpose | Patients of chronic constipation without defecation desire will be orally administered elobixibat 10 mg once daily before meals for 4 weeks. The primary endpoint of the pre/post comparative study will be the percentage of improvement in loss of defecation desire from Week 2 of the observation period at Week 4 of the treatment period. |
| Target number of subjects | 40 cases (as this registration case) |
| inclusion criteria | **selection criteria**  Patients who meet all of the following   - at the time of provisional registration  1. Patients diagnosed with chronic constipation according to the Rome IV criteria for the diagnosis of chronic constipation 2. Age: 20 years or older (at the time of obtaining consent) 3. Gender: Any 4. outpatients 5. Patients for whom written consent can be obtained 6. Patients who can record defecation, etc. in the patient diary   At the time of registration: Dosing start criteria  Patients with the following  "loss of defecation desire*" in the second week of the observation period (1 week before the start of the treatment period) *  *" loss of defecation desire " refers to patients whose "presence or absence of defecation desire " on the patient questionnaire was "4. almost never” or “5. never".  **Exclusion criteria**  **At the time of temporary registration**  Exclude patients with any of the following conditions   1. Patients with organ-related constipation or suspected of having organ-related constipation 2. Patients with or suspected of having functional ileus 3. Patients with or suspected of having inguinal hernia 4. Patients with a history of open abdominal surgery within 12 weeks prior to obtaining consent (excluding appendicitis resection) 5. Patients with a history of surgical or endoscopic procedures related to gallbladder resection and papillotomy 6. Patients with complications of malignancy   However, patients who have undergone radical surgery or who have completed chemotherapy or radiotherapy may be registered.   1. Pregnant women, lactating women, women who may be currently pregnant, or patients who cannot give consent to use contraception while participating in the study 2. Patients with serious renal, hepatic, or cardiac disease 3. Patients with drug allergy to the study drug 4. Patients who are participating in other clinical studies, or who have participated in other clinical studies within 4 weeks prior to obtaining consent   However, observational studies are excluded.   1. Other patients who are judged by the principal investigator or sub-investigator to be inappropriate for this study.   At the time of registration: Dosing start criteria  (1) Patients who increased the dose of concomitantly restricted drugs during the observation period  (2) Patients who used concomitantly prohibited drugs during the observation period |
| Name and dosage of the study drug | Elobixibat  Dose: Start with 10 mg (2 tablets) of elobixibat, and then can be increased or decreased (5 mg or 15 mg) according to symptoms. |
| Method of administration, observation and duration of administration | The study will consist of a 2-week observation period and a 4-week treatment period.  During the treatment phase, the study drug is administered orally once daily before meals for 4 weeks. |
| evaluation item | **Effectiveness**  Primary endpoint.  Percentage of improvement in loss of defecation desire in the fourth week of the treatment period from the second week of the observation period (patient questionnaire)  Secondary endpoints: The following items will be evaluated and their relevance will be examined.  (1) Changes in the following items in each week of the treatment period and comparison of Week 4 of the treatment period with Week 2 of the observation period   1. Presence of defecation desire: patient questionnaire 2. Satisfaction with defecation desire: a patient questionnaire 3. Satisfaction with straining: patient questionnaire 4. Degree of straining: patient diary 5. Presence of a sense of incomplete evacuation: patient diary 6. Satisfaction with treatment: patient questionnaire 7. Spontaneous bowel movement (SBM) frequency 8. Complete Spontaneous Bowel Movement (CSBM) frequency 9. Stool hardness based on the Bristol Stool Form Scale     (2) Comparison of the following items at Week 4 of the treatment period with Week 2 of the observation period  1. Constipation score: CSS  2. JPAC-QOL score  3. absolute value and percent composition of bile acid concentration in feces  (3) Changes in the time from taking elobixibat to defecation each week during the treatment period  (4) Consideration of the relationship between the evaluation items  **safety**  Incidence rate of diseases, etc. |
| contact information (for inquiries) (e.g. corporate phone number) | Principal Investigator: Department of Palliative Medicine, Yokohama City University Hospital  Takaomi Kessoku  TEL:045-787-2800 (representative)  Research secretariat: Yokohama City University Hospital  Next Generation Clinical Research Center  　　　TAKEMOTO Emiko WATANABE Orie  TEL: 045-370-7994 FAX: 045-342-5875 |
| Rules and Regulations to be observed | All persons involved in this research will conduct it in accordance with the "Clinical Research Act", the "Enforcement Regulations of the said Act and other related notifications", in accordance with the "Declaration of Helsinki of the World Medical Association", which all medical research involving human subjects should comply with. |

# **1. Purpose of this study**

Patients with chronic constipation will be orally administered elobixibat 10 mg once daily before meals for 4 weeks. A before-and-after study will be conducted, and the rate of improvement in loss of defecation desire from the second week of the observation period in the fourth week of the treatment period will be the primary endpoint.

# **2. background and scientific rationale for the study design**

Elobixibat specifically inhibits IBAT/ASBT (ileal bile acid transporter/apical sodium-dependent bile acid transporter), which is a transporter involved in the reabsorption of bile acids at the terminal ileum [1]. It was approved for the treatment of chronic constipation in Japan in January 2018. It is thought that the IBAT inhibitory action of elobixibat increases the amount of bile acids reaching the colon by inhibiting bile acid reabsorption, thereby promoting water secretion into the lumen of the large intestine and gastrointestinal motility. In addition, bile acids are expected to have a restorative effect on defecation desire because they have an effect to lower the rectal sensory threshold, which is an objective index of defecation desire [2,3].

In 2020, a web-based questionnaire-based survey conducted at Yokohama City University reported that patients with chronic constipation had a significantly higher rate of loss of defecation desire than healthy adults, with about 60% of patients losing their defecation desire [4]. Patients whose defecation desire were improved by treatment had higher treatment satisfaction than those whose defecation desire were not improved [4], suggesting that the presence or absence of bowel movements in the treatment environment is related to treatment satisfaction and the importance of defecation desire in the treatment of constipation. However, in actual clinical practice, it has not been investigated whether elobixibat, which increases bile acids in the colon, has a restorative effect on defecation desire and how long it takes for recovery.

In a double-blind, placebo-controlled, 2-week study, elobixibat improved various symptoms in Japanese patients with chronic constipation, such as the number of spontaneous bowel movements (SBM), the number of complete spontaneous bowel movements (CSBM), the time to the first spontaneous bowel movement, and stool hardness, [5]. Safety and efficacy have also been confirmed in a single-arm study without a control group for 52 weeks [5]. None of these studies examined recovery of defecation desire.

In this study, we will investigate the improvement rate of defecation desire and symptoms of defecation difficulty in patients with chronic constipation, based on the improvement rate of bowel movements after 4 weeks of treatment with elobixibat.

## 2.1 Target diseases

Chronic constipation is a frequent functional disorder encountered in daily clinical practice with a prevalence of 2-27% in Japan. It is more common in women than in men, and its prevalence increases with age in both sexes [6]. In addition, a decrease in quality of life (QOL) has been reported, including a significant decrease in daily activity and work productivity compared to patients without chronic constipation [7]. In addition, many patients with chronic constipation are said to be less satisfied with conventional treatment methods, and it has been reported that they have more subjective symptoms such as abdominal distension and dyspepsia as well as frequency of defecation and hardness of stool [8].

Understanding the unpleasant symptoms of this "difficulty" and improving constipation symptoms, including recovery of defecation desire, will lead to an increase in QOL.

## 2.2. Standard treatment

2.2.1 History of the standard treatment that has been implemented so far, etc.

Pharmacotherapy with laxatives is commonly used for chronic constipation in which symptoms do not improve sufficiently despite guidance on diet, lifestyle, and defecation habits. Two types of laxatives, magnesium oxide and stimulant laxatives, are widely used in clinical practice. Magnesium oxide is the most commonly prescribed drug, and regular monitoring of serum magnesium concentration is necessary for use in elderly patients and patients with impaired renal function such as those with chronic kidney disease.

On the other hand, although stimulant laxatives have powerful effects, they should be used only as a general rule, because of concerns about dependence and drug resistance due to continuous use. Magnesium oxide, which can be finely adjusted in dose and is highly safe, is therefore used as the first-line drug. However, as mentioned above, there is a risk of hypermagnesemia not only in patients with impaired renal function and patients taking long-term high doses, but also in rare cases with normal renal function. Therefore, it is recommended to monitor serum magnesium levels at 3-6 month intervals during long-term use [9].

2.2.2 Current standard of care

Pharmacotherapy for chronic constipation is considered to be one of the standard treatments for constipation along with dietary guidance and lifestyle guidance. However, as mentioned above, there are problems such as side effects and tolerance / addiction during long-term use for various drugs that are the first choice [10-12].

In recent years, the number of drugs for the treatment of chronic constipation has increased due to the emergence of linaclotide and lubiprostone preparations with a high level of evidence. In addition, the Ministry of Health, Labour and Welfare (MHLW) has issued five preparations, linaclotide, lubiprostone, elobixibat hydrate, macrogol 4000 combination, and lactulose, for use in patients with inadequate efficacy of existing constipation medications [13,14]. The treatment situation has changed significantly.

## 2.3. Study treatment

The study drug elobixibat was approved for the manufacture and sale as a treatment for chronic constipation (excluding constipation due to organic disease) in January 2018. By inhibiting the bile acid reabsorption transporter in the terminal ileum, elobixibat increases bile acids in the colon and promotes water and electrolyte secretion as well as gastrointestinal motility. Since Clozapine is only slightly absorbed into the body and does not enter the bloodstream to exert its effects, there is little concern about drug interactions with concomitant medications.

As risks of the study treatment, abdominal pain and diarrhea are described in the package insert as the main adverse reactions. In a domestic clinical study of 631 patients with chronic constipation, adverse drug reactions including abnormal laboratory values occurred in 292 (46.3%) patients, and the major adverse drug reactions were abdominal pain in 120 (19.0%) patients and diarrhea in 99 (15.7%) patients.

As a benefit of the study treatment, the percentage of improvement in defecation desire after 4 weeks of treatment will lead to the confirmation of recovery of defecation desire and the improvement of symptoms of dysuria, which will lead to the improvement of QOL and treatment satisfaction.

## 2.4. Study design and primary endpoints

This was a multicenter, single-arm, open-label, before-and-after study in patients with chronic constipation.

[Primary endpoint] Percent improvement in loss of defecation desire from Week 2 of the observation period at Week 4 of the treatment period (patient questionnaire)

[Basis for setting]

The EMA guideline states that it is important to assess quality of life [15], and a web-based questionnaire survey in 2020 reported that patients with chronic constipation had a higher rate of loss of defecation desire than healthy adults, and that patients whose defecation desire improved with treatment had a higher level of treatment satisfaction [4]. We hypothesize that elobixibat increases the amount of bile acids that enter the lumen of the large intestine, and that these bile acids lower the rectal sensory threshold, which leads to the recognition of defecation desire.

In this study, "confirmation of recovery of loss of defecation desire ", which is considered to be related to treatment satisfaction, was the primary endpoint.

In the previous literature [5], improvement in the number of spontaneous bowel movements, etc., was observed after 2 weeks of treatment, and since elobixibat is effective in improving constipation at an early stage, we believe that early recovery of bowel movements can be expected in actual clinical practice. In this study, we set "confirmation of recovery of defecation desire " at 4-week administration, which is the usual interval between visits.

## 2.5 Significance of this study

The primary endpoint of the study was "confirmation of recovery of loss of defecation desire ".

# **3. Study drug information**

## 3.1. Study drug

Generic name: Elobixibat hydrate

Product name: Goofis® Tablets 5mg

Storage method: room temperature storage

Name of manufacturing and marketing company: EA Pharma Co.

Indications: Chronic constipation (excluding constipation caused by organic diseases)

Dosage and Administration (Method of Use): The usual adult dosage of elobixibat is 10 mg orally once daily before meals. The dosage may be adjusted according to symptoms, but the maximum dosage is 15 mg per day.

*Formulation* (appearance): Pale yellow, circular, film-coated tablets

## 3.2 Management of study drugs

The study drug will be Goofis® Tablets 5 mg. Refer to the attached document for prescription and administration.

## 3.3 Ensuring the quality of study drugs

Refer to the attached document to ensure quality.

# **4. Criteria and definitions used in this study**

|  | (data) Item | Contents | Appendix / Patient diary |
| --- | --- | --- | --- |
| Criteria | Diagnosis of chronic constipation | Diagnostic criteria for functional constipation in Rome IV (Drossman, DA. Hasler, WL. Gastroenterology. 2016; 150: 1257-1261.) | Appendix E |
| definition | Spontaneous bowel movement (SBM) | Defecation that occurs without transanal laxative, enema or disimpaction: The number of "defecation that occurs without suppository, enema or disimpaction" in 7 days. Defecation within 24 hours after using a suppository or enema and defecation during disimpaction are not counted in the "number of spontaneous defecations". | Patient diary |
| definition | Complete Spontaneous Bowel Movement (CSBM) | Defecation occurring without residual sensation and without transanal laxatives/enema or removal of stool | Patient diary |
| Criteria | Satisfaction with defecation desire, bearing down, and treatment | On a scale of one to five.  1. very satisfied, 2. somewhat satisfied, 3. average  4. somewhat dissatisfied, 5. very dissatisfied | Appendix A: Patient Questionnaire |
|  | Defecation desire | On a scale of one to five.  1. always available, 2. usually available, 3. a little, 4. hardly, 5. not at all | Appendix A: Patient Questionnaire  Patient diary |
|  | Bearing down to defecate | On a scale of one to five.  1. Not pushing at all, 2. pushing a little, 3. Pushing to some extent, 4. Pushing hard, 5. pushes very hard | Patient diary |
|  | Symptom severity of constipation | Constipation Score (CSS):.  Evaluation of 8 items (frequency of defecation, difficulty in defecation, feeling of residual defecation, abdominal pain, time required for defecation, presence or absence of assistance in defecation, number of times when stool was not passed even after going to the toilet/24 hours, duration of illness with defecation disorder: year) on a 30-point scale | Appendix B |
|  | Classification of stool properties | Bristol Stool Shape Scale (BS Score): a global standard for assessing stool properties. 7-point scale for each bowel movement | Appendix C |
|  | QOL assessment | The Patient Assessment of Constipation Quality of Life (JPAC-QOL): A Disease-Specific Quality of Life Rating Scale for Chronic Constipation | Appendix D |

# **5. Subject selection policy**

## 5.1. Selection criteria

Patients who meet all of the following

At the time of temporary registration

- 1. Patients diagnosed with chronic constipation according to the Rome IV criteria for the diagnosis of chronic constipation
  2. Age: 20 years or older (at the time of obtaining consent)
  3. Gender: Any
  4. Outpatients
  5. Patients for whom written consent can be obtained
  6. Patients who can record defecation, etc. in the patient diary

　　At the time of registration: Dosing start criteria

Patients with the following

Patients with loss of defecation desire* in the second week of the observation period (1 week before the start of the treatment period)

*"Loss of defecation desire " refers to patients whose "presence or absence of defecation desire " on the patient questionnaire was "4. almost never, 5. never".

(Basis for setting: At the time of temporary registration)

1. We have used the internationally widely used Rome IV as the diagnostic criteria for chronic constipation.
2. To participate in the study of their own free will, participants had to be at least 20 years of age to legally give their personal consent.
3. Set in order to maximize the number of patients we will be able to recruit.

(4) Constipation is mainly treated as an outpatient condition.

1. Set in compliance with the spirit of the Declaration of Helsinki.
2. Set in order to properly evaluate the efficacy of study drugs.

　　(Basis for setting: At the time of registration)

　　The primary endpoint of 　　the study was set to be assessed correctly.

## 5.2. Exclusion criteria

Patients who meet any of the following conditions should be excluded

At the time of temporary registration

1. Patients with organic constipation or suspected of having organic constipation
2. Patients with or suspected of having functional ileus
3. Patients with or suspected of having inguinal hernia
4. Patients with a history of open abdominal surgery within 12 weeks prior to obtaining consent (excluding appendicitis resection)
5. Patients with a history of surgical or endoscopic procedures related to gallbladder resection and papillotomy
6. Patients with complications of malignancy

However, patients who have undergone radical surgery or who have completed chemotherapy or radiotherapy may be registered.

1. Pregnant women, lactating women, women who may be currently pregnant, or women who use contraception while participating in the study.

Patients who do not give their consent to be

1. Patients with serious renal, hepatic, or cardiac disease
2. Patients with a history of drug allergy to this study drug
3. Patients who are participating in other clinical research, or who participated in other clinical research within 4 weeks before obtaining consent

Patients who participated in the study but not in observational studies

1. (3) Any other information that the principal investigator or sub-investigator deems inappropriate for conducting this research.

disabled patient

At the time of registration: Dosing start criteria

(1) Patients whose doses of concomitantly restricted medications were increased during the observation period

(2) Patients who used concomitantly prohibited drugs during the observation period

(Basis for setting: At the time of temporary registration)

- 1. ~5) Set because they may affect the efficacy of the study drug.

(6) to (9) were set to ensure the safety of the subjects.

(⑩) Set to eliminate any effects on ethical considerations and the evaluation of study drugs.

(⑪) In addition to the above, it is judged that the subject of this research is inappropriate from the scientific aspect, ethical viewpoint, etc.

It was set assuming the case where the data is used for the first time.

(Basis for setting: At the time of registration)

　　　　　　　　(1) (2) The primary endpoint of this study was set to be evaluated correctly.

# **6. Research plan**

## 6.1 Research Design

A multicenter, single-arm, open-label, before-and-after study

## 6.2. Target number of cases

　　40 cases (as the number of registered cases)

## 6.3. Research period

Registration period: from the date of jRCT publication (date of notification by the Minister of Health, Labour and Welfare) to July 31, 2022

Study period: From the date of publication of the jRCT (date of notification to the Minister of Health, Labour and Welfare) to March 31, 2023 (approximately 2 years)

Planned duration of subject participation: up to 9 weeks from obtaining consent

Observation period: 2~4 weeks

Treatment period: 4 weeks (maximum 5 weeks)

## 6.4 Facility Registration and Case Registration

Institutional registration shall be done by central registration at the data center.

Case registration will be possible from the implementing medical institution that has completed the facility registration.

6.4.1. Data center

JORTC Data Center

TEL: 03-5604-9850 FAX: 050-3737-9544

Mail: r-lodd@dc.jortc.jp

Office hours: 10:00-16:00 on weekdays (except national holidays, Saturdays, Sundays, and year-end and New Year holidays)

6.4.2. Facility registration

The principal investigator of each medical institution shall obtain approval for the implementation of the study from the administrator of the medical institution after approval by the accredited clinical research review committee, and then send a copy of the administrator's approval letter to the data center. The data center shall register the facility and notify the principal investigator by sending a notice of completion of facility registration.

6.4.3 Enrollment of Subjects

　 The principal investigator or sub-investigator assigns a subject identification code for all subjects who have given consent. The eligibility test will be conducted, and at the time of provisional registration, registration-related information will be entered into the eCRF: Registration Form and saved. If a subject is judged to be "eligible", a case registration number will be displayed on the EDC system screen. The case registration number is recorded in the subject screening list.

At the time of registration, confirm that the patient meets the criteria for starting treatment, and enter and save the registration-related information in the eCRF: Registration Form. If the case is determined to be "Full Registration", the word "Full Registration" will be displayed on the EDC system screen. Prescribe the study medication to the enrolled subjects. Record the date of "Full Registration" in the Subject Screening List. For cases that do not meet the dosing start criteria, enter and save the pre-start dropout information on the eCRF: Full Registration form.

　　Subjects who do not meet the criteria for starting dosing and who drop out before starting dosing may be re-enrolled. In this case, consent should be obtained again from the subject, and eligibility tests should be conducted and confirmed.

6.4.4 Allocation Method and Allocation Adjustment Factors

　　Not applicable.

6.4.5 Method of blinding

　Not applicable.

6.4.6 Determining the need for and procedures for opening emergency key codes

　　　Not applicable.

6.4.7 Opening the Study drug Allocation Code Table

Not applicable.

## 6.5. treatment plan

6.5.1. protocol treatment

Description.

consent acquisition

Observation period: 2~4 weeks

Patient diary entry

Confirmation of dosing criteria

Eligibility check

Provisional registration

Treatment period 4 weeks

Writing patient diary/patient questionnaire

Observation period

Treatment period

final registration

6.5.1.1. observation period

Observation period: 2~4 weeks

The subject will keep a patient diary during the observation period.

6.5.1.2. treatment period

After the end of the observation period, administration of the study drug will be started on the day of enrollment after confirming that the patient meets the criteria for starting the drug. Patient diary entries and questionnaires should be completed throughout the treatment period.

Elobixibat is administered orally once daily before meals for 4 weeks.

The starting dose of elobixibat is 10 mg (2 tablets), and the dose can be increased or decreased (5 mg: 1 tablet or 15 mg: 3 tablets) according to the symptoms.


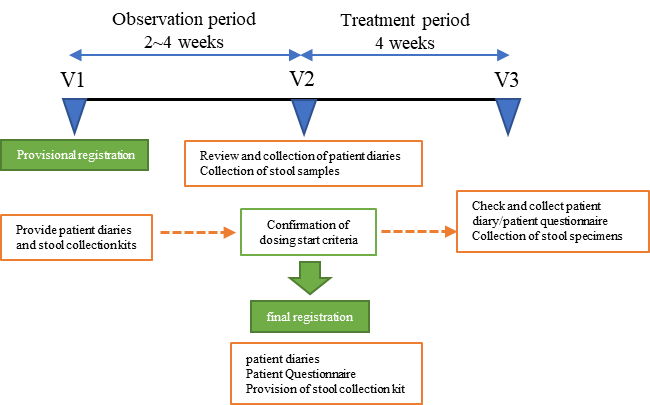


6.5.2. Concomitant medications/adjunct therapy

6.5.2.1 Restricted Concomitant Drugs and Therapies: Observation Period to End of Treatment Period (Final Dose)

Chronic constipation medications (including over-the-counter medications, supplements, etc.) and therapies used prior to enrollment should not be changed in principle until the end of the treatment period. However, dose reduction or discontinuation is acceptable. The use of enemas, suppositories, and stool extraction is permitted only when adequate defecation is not observed.

6.5.2.2 Concomitant medications prohibited: from observation period to end of treatment period (last dose)

The use of the following drugs, which may affect this study, is prohibited.

Bile acid transporter inhibitors other than study drugs

Bile acid preparations (ursodeoxycholic acid, kenodeoxycholic acid, dehydrocholic acid)

Antacids containing aluminum (sucralfate hydrate, aldioxa, etc.)

Cholestyramine, Cholestymid

6.5.2.3 Concomitant medications: during treatment (until last dose)

Drugs that should be used with caution are listed below.

-Digoxin

Dabigatran etexilate methanesulfonate

Midazolam

6.5.3. Instruction of subjects

At the time of provisional enrollment, the principal investigator or sub-investigator will hand out the patient diary to the subjects and instruct them on how to write it and the following precautions. For patients who have been enrolled in the study, the patient diary and patient questionnaire will be filled out, the method of taking the study medication will be explained, and the following precautions will be explained again.

1. Patients should not change their lifestyles, such as diet and exercise, during the study period.
2. Instruct the patient to complete the patient questionnaire provided at the time of this registration on a weekly basis.
3. Patients should be instructed to keep a patient diary from the beginning of the observation period to the end of the study medication, and to record the status of defecation, the status of taking the study medication, and the status of using suppositories and enemas.
4. When visiting another hospital, patients should inform the physician of your participation in the study. They should inform the principal investigator or sub-investigator in advance of the visit to another hospital and of any new prescription medications, tests, or treatments.
5. Patients must come to the hospital on the prescribed day, and if they cannot come to the hospital due to unavoidable circumstances, you must contact the hospital in advance.
6. Patients should tell the principal investigator or sub-investigator about any medications, supplements, or treatments you are taking, including prescription medications from other hospitals or over-the-counter medications purchased at pharmacies.
7. The study drug should be taken before meals once a day from the day of administration according to the instructions of the principal investigator or sub-investigator.
8. If patients miss a dose, take it on the same day if possible.
9. Be sure to bring the remaining medication, patient diary, and patient questionnaire to the next visit.
10. Bring the stool samples collected at the time of visit (V2 , V3).
11. When you come to the hospital (V2, V3), you should fast from 8 hours before the test because the blood test will be performed.
12. Women of childbearing potential should use an appropriate method of contraception from the time of obtaining consent until the end of the treatment period.

6.5.4. Dose reduction and withdrawal criteria

not applicable

6.5.5. Criteria for volume increase and resumption

　　not applicable

**7. Observation, examination, investigation and evaluation items**

## 7.1 Schedule

|  | Obtaining  consent | Observational periods | | Treatment periods |
| --- | --- | --- | --- | --- |
|  |  | V1 | V2 | V3/EOT |
| Week |  | Provisional registration | Before treatment/final registration | 4 weeks |
| Allowance |  | － | After provisional registration 2~4 weeks | ±7日 |
| Obtaining consent | ○ |  |  |  |
| Selection/exclusion criteria |  | ○ | 〇 |  |
| Subject background |  | ○ |  |  |
| Vital sign ^a^ |  |  | ○ | ○ |
| Subjective/objective symptoms findings |  |  | ○ | ○ |
| hematological tests |  |  | 〇 | ○ |
| Biochemical tests |  |  | 〇 | ○ |
| Provisional registration |  | ○ |  |  |
| Final registration/ Dosing start criteria |  |  | ◎ |  |
| Blood and stool for exploratory research samples* collection |  |  | ● | ● |
| Research drug prescription |  |  | ○ |  |
| Checking medication status |  |  |  | ○ |
| Investigation of concomitant medications |  | ○ | ○ | ○ |
| Investigation of adverse events |  |  |  |  |
| Patient Diary ^b^ |  |  |  |  |
| Patient Questionnaire^c^ |  |  | 〇 |  |
| questionnaire survey ^d^ |  |  | 〇 | 〇 |

*: Provide stool collection kits for V1 and V2.

a: Blood pressure Pulse

b: Provide patient diary (for observation period) to V1, check and collect from V2.

　 Provide patient diary (for therapeutic phase) to V2, check and collect to V3.

^c^： V2 Patient questionnaire will be administered on arrival at the hospital.

Patient questionnaires for the treatment period are provided to V2, and checked and collected by V3.

^d^: JPAC-QOL and constipation score (CSS) will be conducted at V2 and V3 visits.

## 7.2 Implementation schedule and evaluation items

7.2.1 Obtaining Consent and Temporary Registration

1. Date of obtaining consent
2. Selection/exclusion criteria
3. provisional registration
4. Subject Background

Sex, age (at the time of obtaining consent), height, weight, history of laparotomy (within the past year)

Previous history: diseases that may affect the efficacy and safety of the target disease (past)

(Within 1 year)

Complications: Major underlying diseases, etc., present at the time of obtaining consent

Duration of illness in chronic constipation

1. Subjective symptoms and other findings
2. Investigation of concomitant medications
3. Explanation and provision of stool collection kits
4. Explanation and provision of patient diary (for observation period)

7.2.2 Pre-administration/enrollment

1. Dosing start criteria
2. Final registration
3. Blood pressure, pulse rate
4. Subjective symptoms and other findings
5. Hematological examination: white blood cell count, hemoglobin, platelet count
6. Biochemical tests: total protein, albumin, AST, ALT, γ-GTP, ALP, total bilirubin, urea nitrogen, creatinine, uric acid, LDL-cholesterol, HDL-cholesterol, total cholesterol, Na, K, Cl
7. Collection of blood and stool samples for exploratory research
8. Investigation of concomitant medications
9. Investigation of adverse events
10. Questionnaire survey: JPAC-QOL Constipation Score (CSS)
11. Patient questionnaire: presence or absence of defecation desire, satisfaction with defecation desire, satisfaction with pushing, satisfaction with treatment
12. Checking and collecting patient diaries: date and time, frequency of defecation, BS score, degree of urgency, presence or absence of residual stool sensation, presence or absence of picked stool, presence or absence of defecation desire, use of suppositories and enemas
13. Explanation and provision of patient diary (for treatment period)/patient questionnaire
14. Explanation and provision of stool collection kits

7.2.3. treatment period 4 weeks / EOT

1. Blood pressure, pulse rate
2. Subjective symptoms and other findings
3. Hematological examination: white blood cell count, hemoglobin, platelet count
4. Biochemical tests: total protein, albumin, AST, ALT, γ-GTP, ALP, total bilirubin, urea nitrogen, creatinine, uric acid, LDL-cholesterol, HDL-cholesterol, total cholesterol, Na, K, Cl
5. Collection of blood and stool samples for exploratory research
6. Investigation of concomitant medications
7. Investigation of adverse events
8. Questionnaire: JPAC-QOL Constipation Score (CSS)
9. Checking and collecting patient questionnaires: presence of defecation desire, satisfaction with defecation desire, satisfaction with pushing, satisfaction with treatment
10. Checking and collecting patient diaries: status of study medication, date and time, frequency of bowel movements, BS score, degree of urgency, presence or absence of residual stool sensation, presence or absence of picked stool, presence or absence of defecation desire, use of suppositories and enemas

## 7.3 Evaluation methods

Not applicable for this study.

# **8. Obtaining consent**

## 8.1 Informed consent

Before the subject participates in the study, the principal investigator or sub-investigator must answer the subject's questions using the consent and explanation documents approved by the authorized clinical research review committee described in the protocol and give the subject sufficient time to consider participation in the study. After confirming that the subject has fully understood the contents of the document, consent for participation will be obtained in writing.

If new information that may affect the subject's intention, which was not expected at the time of obtaining consent, is obtained during the study, the consent / explanatory document will be revised promptly. Explain using the consent / explanatory document approved by the Accredited Clinical Research Review Board, confirm the intention to participate in the research again, and obtain consent. In addition, if there is a change in the research content, etc., re-consent will be obtained in the same way.

The subject will be informed that he or she can withdraw consent at any time if he or she wishes, even after consenting to participate in the study. When withdrawing consent, the principal investigator or sub-investigator will consult with the subject as much as possible, confirm the reason for withdrawing consent, and explain the treatment method for constipation after withdrawing consent.

The subject's withdrawal of consent will then be obtained by written withdrawal of consent whenever possible.

## 8.2 Response to consultations, etc., from subjects and related persons

The principal investigator or sub-investigator will respond to consultations from subjects and their related persons. If the method of response is unclear, it will be done after consultation with the research secretariat according to the content of the consultation.

## 8.3 Obtaining Informed Consent from a Consignee, etc.

Not applicable because the subjects of this study were 20 years of age or older and able to provide written consent for participation in this study.

## 8.4. When obtaining an informed assent

Not applicable for this study.

## 8.5 Cases in which it is not necessary to obtain consent from subjects, etc.

Not applicable for this study.

# **9. Evaluation items**

## 9.1 Primary endpoints

　　Percent improvement in loss of defecation desire in the fourth week of the treatment period from the second week of the observation period (patient questionnaire)

　　[Definition.

One week before the start of administration of the research drug is the second week of the observation period.

The 4th week from the start of administration of the research drug is the 4th week of the treatment period. However, if a patient comes to the hospital with the permitted allowance of -7 days, the 3rd week will be considered the 4th week of the treatment period.

For discontinued patients, if the study drug has been taken for 1 week or longer, the evaluation at the time of discontinuation should be considered to supplement the missing data.

[Basis for setting]

The EMA guideline states that it is important to assess quality of life [15], and a web-based questionnaire survey in 2020 reported that patients with chronic constipation had a higher rate of loss of defecation desire than healthy adults, and that patients whose defecation desire improved with treatment had a higher satisfaction with treatment [4]. We hypothesize that elobixibat increases bile acid influx into the lumen of the large intestine, and that the decrease in rectal sensory threshold due to this bile acid causes recognition of defecation desire.

In this study, "confirmation of recovery of loss of defecation desire ", which is considered to be related to treatment satisfaction, was used as the primary evaluation.

The items were.

In the previous literature [5], improvement in the number of spontaneous bowel movements, etc., was observed after 2 weeks of treatment, and since elobixibat is effective in improving constipation at an early stage, we believe that early recovery of defecation desire can be expected in actual clinical practice. In this study, we set "confirmation of recovery of defecation desire " at 4-week administration, which is the usual interval between visits.

## 9.2 Secondary endpoints

The following items will be evaluated, and their relevance will be discussed.

1. Changes in the following items at each week of the treatment period and comparison of week 4 of the treatment period with week 2 of the observation period

1. Presence of defecation desire: patient questionnaire

2. Satisfaction with defecation desire: patient questionnaire

3. satisfaction of straining: patient questionnaire

4. Degree of straining: patient diary

5. Presence of a sense of incomplete evacuation: patient diary

6. Treatment satisfaction: Patient Questionnaire

7. Spontaneous bowel movement (SBM) frequency

8. Complete Spontaneous Bowel Movement (CSBM) frequency

9. Fecal hardness based on the Bristol stool form scale

1. Comparison of the following items at Week 4 of the treatment period with Week 2 of the observation period

1. Constipation score: CSS

2. JPAC-QOL score

3. Absolute value and percent composition of bile acid concentration in feces

③ Time from taking elobixibat to defecation in each week of the treatment period.

④ Consideration of the relationship between the evaluation items

## 9.3 Safety evaluation items

　　Incidence rate of diseases, etc.

# **10. Exploratory research**

In this study, blood and stool samples will be collected as samples.

To investigate the effect of changes in blood and fecal bile acid concentrations induced by study drugs on defecation desire

(3) Secondary use of samples and information In the case of secondary use of stored samples (blood and stool), follow "11.3 Secondary use of samples and information".

## 10.1 Time of sample collection and destination

Each sample shall be collected at the time shown in "7.1. Schedule". In accordance with the procedure manual to be prepared separately

Each processed sample is transported to the Department of Hepatobiliary and Pancreatic Gastroenterology, Yokohama City University. After transport, stools are incubated at 0°C

The blood should be stored appropriately in a freezer at -20°C or below.

## 10.2 Management, storage and disposal of samples

Each sample will be stored under the case registration number issued after provisional registration in this study.

Samples will be stored with the specimen control chart. Department of Hepatobiliary and Pancreatic Gastroenterology, Yokohama City University, Yokohama, Japan

The retention period for residual samples stored in the repository shall be five years.

Stored samples should be disposed of after the 5-year storage period, unless there is a specific reason not to do so.

If consent is withdrawn, the anonymized number, etc. will be deleted and the data will be disposed of appropriately.

## 10.3 Withdrawal of consent for use of samples

## If the subject withdraws consent to use the provided sample, the sample will be disposed of / discarded and will not be used in the study. However, this does not apply if the research results have already been announced at the time of withdrawal of consent.

## If the measurement / analysis has already been performed, there is no obligation to discard the result.

The Principal Investigator shall confirm that the following have been implemented:

1. The subject's withdrawal of consent for the use of the sample provided is reported immediately. If samples collected from the subject are stored at the implementing medical institution, they must be immediately identified and disposed of/disposed of, and this must be recorded.
2. That the institution storing and measuring the samples was immediately informed of the withdrawal of consent, that the samples were disposed of/destroyed, and that this was recorded.
3. The subject and Principal Investigator must be informed that the sample has been disposed of/destroyed.

# **11. Storage of samples and information and storage period**

## 11.1 Storage, retention period and disposal of samples

In this study, blood and stool samples will be collected for exploratory research. The procedures for storage, disposal, etc. will be in accordance with the procedures described in "10. Exploratory research.”

## 11.2 Storage management and storage period of information

The principal investigator should keep paper-based information other than the medical records listed below in a lockable vault under strict control. Information on electronic media will be stored on a personal computer or an electromagnetic storage medium such as a USB memory stick that is independent of the hospital LAN or the Internet, with a password set, and will be stored securely in a lockable vault when not in use.

When connecting computers used in this research to the Internet or hospital LAN, be sure to use appropriate security measures such as anti-virus software, and do not connect to public LANs or other lines to which many people can connect. The storage period is five years after the completion of the research. Documents and records that have passed the storage period are disposed of with the utmost care to prevent leakage of personal or confidential information. Paper media will be shredded and disposed of. Other media will be anonymized and disposed of by deletion or other appropriate methods.

Matters specifying the subject (Correspondence table)

Matters related to medical treatment and examination of subjects

Research implementation plan, implementation plan, original materials, etc.

Explanation documents and consent documents for subjects

Consent form (original with signature)

The main evaluation items, the summary report and the summary report

Notification of the results of the review received from the Authorized Clinical Research Review Committee, etc.

A copy of the report to the Minister of Health, Labour and Welfare other than the implementation plan

Monitoring documents

Contract for the conduct of specific clinical research

Other important documents related to the research, as specified by the principal investigator

## 11.3 Secondary use of samples and information

There is a possibility that the researchers involved in this research may use the information and research data obtained in this research for different research purposes or provide them to other research organizations. In such a case, a new research protocol will be prepared, and the research will be conducted after approval is obtained from the Ethics Review Committee that should hear opinions on the research. Informed consent should be given in an appropriate manner according to the nature of the research.

## 11.4 Use as a biobank for samples and information

　Not applicable.

# **12. Handling diseases, etc.**

## 12.1 Definition of diseases, etc.

An adverse event is any unfavorable or unintended injury or illness or sign thereof (including abnormal laboratory values) occurring in a subject, whether or not causally related. It includes any exacerbation of a pre-existing disease (not including the primary disease) during the study period.

Among adverse events, in addition to illness, disability, death, or infection, "diseases, etc." refers to abnormalities in clinical laboratory values or symptoms that are suspected to be caused by the conduct of the clinical research. Diseases, etc. attributable to the conduct of clinical research are those that have a causal relationship with the drugs used in the research or with the research procedures. The causal relationship shall be determined by the principal investigator, sub-investigator, or principal investigator based on "12.3 Causal relationship with research.

## 12.2 Evaluation of diseases, etc.

In this study, the degree (severity) of diseases, etc. occurring from the time of enrollment to the end of taking the study drug will be evaluated as follows.

1) Mild: Can be continued with no treatment

2) Moderate: administration can be continued with some treatment

3) Severe: Conditions for which administration should be discontinued or stopped, etc.

## 12.3. Causal relationship with research

Causality will be assessed according to the following criteria.

**Causal relationship present or undeniable** - judgment is made according to the following criteria regardless of whether the event in question is known or unknown to occur by the study or intervention treatment.

- - Reasonable or reasonably likely to be attributable to the research or interventional treatment
  - There is a temporal relationship with the study
  - No other cause can be shown, and a causal relationship with the study cannot be ruled out.

**No causal relationship** - to be determined according to the following criteria

- - not reasonably attributable to the research or interventional treatment.
  - temporal relationship cannot be shown
  - Other causes can be shown

## 12.4. Causal relationship to the study drug

Causality is evaluated according to the following criteria. "Causal or undeniable" is to be considered an adverse effect.

**Causal or undeniable** - Regardless of whether the event is known or unknown to be caused by the study drug, the following criteria should be followed

- - Reasonable, or reasonably likely, to be caused by the study drug
  - There is a time relationship between the study drug
  - No other cause can be shown, and a causal relationship with the study drug cannot be ruled out.

**No causal relationship** - to be determined according to the following criteria

- - not reasonably attributable to the study drug.
  - Cannot show temporal relationship.
  - Other causes can be shown.

## 12.5. Predictability

　　Judgment of predictability is made based on the package insert, interview form, etc., as the research drug is used within the range of approved indications.

[Anticipated diseases, etc.]

In domestic clinical studies up to the time of approval, adverse reactions including abnormal laboratory test results were observed in 292 of 631 cases (46.3%). The major adverse reactions were abdominal pain in 120 cases (19.0%) and diarrhea in 99 cases (15.7%). Other adverse reactions were as follows

|  | 5% or more | 1 to less than 5 | Less than 1 percent |
| --- | --- | --- | --- |
| liver ^注1）^ |  | abnormal liver function test  (ALT (GPT) increased,)  (AST (GOT) increased) |  |
| psychoneurotic system |  |  | Headache, floating dizziness |
| circulatory organ |  |  | a glow |
| digestive organs | Abdominal pain (19.0%), diarrhea (15.7%), lower abdominal pain, abdominal distention | Nausea, Upper abdominal pain, Abdominal discomfort, Soft stool | flatulence, thirst, urgency of stool, dyspepsia  Vomiting, Abnormal gastrointestinal sounds, Constipation, Stomatitis |
| hypersensitivity ^注2）^ |  |  | Urticaria, rash |
| blood |  |  | Increased eosinophil count, anemia  Increased Vitamin E |
| the others |  | CK (CPK) increased | dysmenorrhea |

(Note 1) Patients should be carefully observed for such symptoms, and if any abnormalities are observed, administration should be discontinued.

(Note 2) If such symptoms occur, administration should be discontinued.

## 12.6 Measures to be taken in the event of the occurrence of diseases, etc.

12.6.1 Measures to be taken for subjects

In the event of an occurrence of diseases, etc., the principal investigator or sub-investigator shall take necessary measures to ensure the safety of subjects.

Appropriate measures, such as discontinuation of treatment or administration of the study drug, will be taken for the subject if necessary. If treatment, etc. becomes necessary, the subject will be informed to that effect.

The principal investigator or sub-investigator must ensure that the instance of diseases, etc., continues at the time of the final observation at the end of the study drug and that the researcher is able to obtain the necessary information.

If the patient is still alive at baseline, follow-up will be conducted thereafter until recovery to baseline status or until the patient is clinically stable.

12.6.2 Evaluation and recording

The principal investigator or sub-investigator should describe in the original documents (medical records, etc.) the name of the disease, the date of occurrence, severity, whether it is serious or non-serious, the reason for judging it to be serious, the details of the treatment, the causal relationship with the study and the study drug, the date of the outcome, the outcome (recovery, mild recovery, not recovered, with sequelae, death, unknown), the reason for terminating the outcome, etc. The reason for terminating the outcome (if unrecovered, with sequelae, or unknown) should be described.

12.6.3 Reporting of serious diseases, etc.

When an occurrence of diseases, etc., that falls under 1 through 7 below occurs, the submitting physician who becomes aware of it shall report it to the principal investigator of the institution to which he/she belongs. The principal investigator who receives the report shall report it to the administrator of the medical institution concerned and notify the principal investigator. Thereafter, depending on the predictability of the diseases, etc., the Principal Investigator shall report to the Accredited Clinical Research Review Committee by the following reporting deadlines after the Principal Investigator becomes aware of it.

1. Death or risk of death
2. Those that require hospitalization or extension of hospital stay at a medical institution for treatment
3. Disability or risk of leading to disability
4. Severe according to 1~3
5. Congenital diseases or abnormalities in later generations
6. 1~5 due to infectious disease
7. Diseases, etc. due to infectious disease that does not fall under item 6 (Non-serious diseases, etc. due to infectious disease)

| classification | diseases, etc. | Predictability, etc. | Deadline for reporting to the Administrator and the Commission |
| --- | --- | --- | --- |
| Non-infectious diseases ^(1)^ | (1) Death | Not relevant | Within 15 days |
|  | (2) Diseases, etc. that require hospitalization or extension of the period of hospitalization at a medical institution for treatment | Unpredictable events, etc. ^(2)^ | Within 15 days |
|  |  | Other than above | Within 30 days |
|  | third obstacle | Unpredictable events, etc. | Within 15 days |
|  |  | Other than above | Within 30 days |
|  | (4) Diseases, etc. that may lead to death or disability | Unpredictable events, etc. | Within 15 days |
|  |  | Other than above | Within 30 days |
|  | (5) Diseases, etc. that is similar in severity to those listed in (2) to (4) above | Unpredictable events, etc. | Within 15 days |
|  |  | Other than above | Within 30 days |
|  | (6) Congenital disease or anomaly in later generations | Unpredictable events, etc. | Within 15 days |
|  |  | Other than above | Within 30 days |
| infectious disease | (7) Diseases caused by infectious diseases, etc. | Unpredictable. ^(3)^ | Within 15 days |
|  | (8) Diseases, etc. listed in (1) to (7) above due to infectious diseases (excluding (7)) | Not relevant | Within 15 days |

- 1. Occurrences of infectious diseases with hematotoxicity shall be classified as "non-infectious diseases".
  2. (i) Those which cannot be predicted from the package insert or interview form of the drug, etc. used in the said specified clinical research, or those which can be predicted, and whose tendency of occurrence cannot be predicted, or whose change in the tendency of occurrence indicates the possibility of the occurrence or spread of health hazards.
  3. Items that cannot be predicted from the "Precautions for Use, etc." in the package insert of the relevant drug, etc.

12.6.4 Reporting of other diseases, etc.

Occurrences of diseases, etc. suspected to be caused by the conduct of specific clinical research (excluding all of those listed above) shall be reported at the time of periodic reporting (every year, counting from the date of submission of the implementation plan to the Minister of Health, Labour and Welfare, within 2 months after the expiration of the relevant period).

Upon learning of the occurrence of diseases, etc., the principal investigator of each medical institution shall notify the principal investigator after reporting to the administrator of the medical institution at the time of the yearly periodic report. (2) The principal investigator shall report the diseases, etc. to the authorized clinical research review committee described in the implementation plan. In addition, the Principal Investigator shall promptly provide information to subinvestigators and said subinvestigators shall promptly report the contents of said information to the administrator of the medical institution.

12.6.5 Response to the Opinion of the Accredited Clinical Research Review Committee

When the committee gives its opinion, the principal investigator shall report to the administrator of the medical institution on the opinion given by the committee. If specific action is required based on the opinions expressed by the committee, the principal investigator shall report to the administrator of the medical institution, including the details of that action.

The Principal Investigator shall provide information to the other Principal Investigators to that effect, and the other Principal Investigators shall promptly report the contents of the information provided to the administrator of the medical institution.

12.6.6 Bug Reporting

Not applicable.

# **13. Discontinuation criteria and procedures**

## 13.1 Discontinuation criteria

If any of the following criteria are met, the subject's participation in the study will be discontinued

1. If the subject requests to withdraw consent
2. If, after provisional registration, it is found that the applicant does not meet the selection criteria or violates the exclusion criteria and is inappropriate as a target
3. If it is found after this registration that the criteria for starting dosing are not met
4. When it is difficult to continue the research due to worsening of symptoms or findings of the diseases, etc.
5. When it is difficult to continue the research due to the occurrence of adverse events
6. In the event of a serious deviation from the research protocol
7. death
8. If you find out you are pregnant
9. When the principal investigator or sub-investigator judges that the continuation of the research is not desirable.

## 13.2 Discontinuation Procedure

If a subject is found to meet the discontinuation criteria, the principal investigator or sub-investigator will explain this to the subject, discontinue the study, and take appropriate measures. If the subject is judged to be ineligible at the time of provisional enrollment, the preparation of a case report is not required, and the study will be terminated without discontinuation. If a subject requests discontinuation of the study, appropriate efforts will be made to ascertain the reason for the discontinuation with full respect for the subject's rights.

1. Discontinuation procedure before taking study medication

If the research is discontinued without taking the research medication, the principal investigator or sub-investigator should enter the date of discontinuation (the date when the decision to discontinue was made) and the reason for discontinuation in the case report form.

1. Discontinuation procedure after taking study medication

Except in cases where the subject's cooperation is not obtained, the investigator will investigate the status of the subjects taking the study drug (if during the administration period) and the occurrence of diseases, etc., as well as the tests and evaluations prescribed for discontinuation. The principal investigator or sub-investigator will enter the date of discontinuation (the date when the decision to discontinue is made) and the reason for discontinuation in the case report form.

The principal investigator or sub-investigator will confirm the presence or absence of diseases, etc., and if diseases, etc., is detected, follow-up investigations will be conducted in principle.

## 13.3 Post-treatment after completion (discontinuation) of protocol treatment

Not applicable.

# **14. Discontinuation of the study**

　　If any of the following situations occurs and the Principal Investigator, the Accredited Clinical Research Review Committee, or the administrator of the implementing medical institution judges that the study should be discontinued, the entire study may be discontinued.

In case of unpredictable serious diseases, etc., which may be detrimental to the whole subject.

Serious violations of or non-compliance with the Act and related laws and regulations or the research protocol are found.

In the event that facts are obtained that undermine or may undermine the ethical validity or scientific rationality

If a significant risk to subjects is identified.

In the event that an opinion is expressed to the Authorized Clinical Research Review Committee

In the event that the Minister of Health, Labor and Welfare requests or recommends that the Company cease operations.

In the case of discontinuation, the principal investigator will report to the principal investigators of all the institutions, the accredited clinical research review committee, and the administrators of the institutions. In addition, he or she will contact the subjects and inform them of the change in the research schedule. In addition, the subject will be promptly informed of the change and the next visit will be arranged to confirm the safety of the subject.

In addition, within 10 days of the date of discontinuation, the Principal Investigator shall prepare a Uniform Form 11 Notice of Discontinuation and notify the Accredited Clinical Research Review Committee. The principal investigator shall prepare Form 4, "Notification of Discontinuation of Specified Clinical Research," and submit it to the Minister of Health, Labor and Welfare. Even when the research is discontinued, the primary endpoint report, the summary report, and the summary report shall be prepared appropriately, and periodic reports and disease reports shall be conducted until the preparation of these reports and the completion of the research.

# **15. Effectiveness and Safety Evaluation Committee**

An efficacy and safety evaluation committee will not be established in this study.

# **16. Statistical analysis**

16.1 Target population for analysis

We define the following three analysis populations.

16.1.1. full analysis set (FAS)

The population shall consist of all enrolled subjects, excluding subjects who fall into any of the following categories.

Subjects with serious violations of selection and exclusion criteria

Subjects who have never received protocol treatment.

Subjects for whom no data other than those collected at the time of enrollment in the study have been collected since enrollment.

16.1.2. target population conforming to the research protocol (per protocol set: PPS)

The population of FAS shall exclude subjects who fall into the following categories

1. Subjects missing the primary endpoint
2. Subjects with problems such as use of contraindicated drugs, low dose compliance, untraceable and missing values, etc.*

*The details of the criteria for acceptance or rejection will be developed after the start of the examination when the acceptance or rejection will be considered.

16.1.3. safety analysis set (SAS)

The population will consist of all subjects who have received at least one dose of the study drug.

16.2 Basis for setting the target number of cases

The target number of patients is 40 cases for this registration.

[Basis for setting]

　　There are no studies investigating the recovery of defecation desire by treating chronic constipation. According to a WEB survey [4] of 2098 Japanese patients conducted at Yokohama City University, the rate of loss of defecation desire (no defecation desire: none + almost none) before treatment for Rome IV chronic constipation and in healthy adults was 57.4% (no defecation desire: none + almost none). 1484/2587 cases) and 8.3% (214/2587 cases), and the rate of defecation desire disappearance in outpatient treatment cases was 27.1% (120/443 cases).

Since the subjects of this study were "patients without defecation desire " who had already been treated for chronic constipation within the scope of usual care, the expected improvement rate of loss of defecation desire was assumed to be 45%, which was lower than 62.1%. The threshold rate of spontaneous change in defecation desire was assumed to be 20%. If the threshold is 20%, the expected value is 45%, the one-sided α is 0.025, and the total number of cases is 37, the power is calculated to be 91.6%, which is statistically significant if the number of cases is 13 or more out of 37. Although within the scope of normal practice, we considered that some patients may drop out of the study after provisional enrollment, so we judged that 40 cases was an appropriate target number of patients for this study.

The number of patients with chronic constipation who were expected to meet the eligibility criteria, including "no defecation desire," at each institution was examined, and it was determined that the number of patients could be accumulated without problems within the study period (the planned enrollment period of approximately six months).

16.3 Statistical analysis methods

16.3.1 Analysis of primary endpoints

16.3.1.1. main analysis

The main analysis will be conducted for FAS, and the exact test for the binomial proportion will be conducted for the percentage of improvement in bowel movements from the second week of the observation period in the fourth week of the treatment period (the percentage of subjects who fell into the categories other than not at all or hardly at all), assuming that the threshold (mother proportion in the null hypothesis) is 20%. A one-tailed (upper-tailed) test will be conducted, with a significance level of 2.5% on each side. Point estimates for the percentage improvement in bowel movements and exact two-sided 95% confidence intervals for the binomial proportions will be calculated. Discontinued cases will contribute only to the denominator in the main analysis (missing data in the numerator will not be supplemented). In addition, as part of the sensitivity analysis, missing data for discontinued cases will be supplemented according to the criteria in "9.1. Primary endpoints". However, completion of missing data in the sensitivity analysis should be performed only when it is judged that completion by evaluation at the time of dose discontinuation is appropriate based on a summary of the missing status of the primary endpoint.

The presence or absence of defecation desire was assessed using a 5-point scale on the patient questionnaire: 1. always, 2. almost always, 3. a little, 4. almost never, 5. never. "The questionnaire was administered by the Ministry of Health, Labour and Welfare. In this study, the frequency of defecation desire was measured using a 5-point scale. In this study, subjects who had no defecation desire were registered.

16.3.1.2. secondary analysis

As a secondary analysis to the primary endpoint, an analysis similar to the primary analysis for PPS will be performed. A subgroup analysis will be performed in which subject background factors are grouped into appropriate categories.

16.3.2 Analysis of secondary endpoints (efficacy)

　　Analysis of secondary endpoints will be performed for FAS.

The significance level of the test is set at 5% two-sided.

The details of the analysis should be described in the statistical analysis plan.

(1) Changes in the following items in each week of the treatment period and comparison of the fourth week of the treatment period with the second week of the observation period

1. Presence of defecation desire: patient questionnaire

Summary statistics of the presence or absence of defecation desire at each week of the treatment period will be calculated and compared with the second week of the observation period by the Wilcoxon signed rank test for the fourth week of the treatment period.

2. Satisfaction with defecation desire: patient questionnaire

Summary statistics of satisfaction with defecation desire at each week of the treatment period will be calculated and compared with the second week of the observation period by the Wilcoxon signed rank test for the fourth week of the treatment period.

3. Satisfaction of straining: patient questionnaire

Summary statistics will be calculated for the level of satisfaction with pushing/bearing down at each week of the treatment period, and the results will be compared with those of the second week of the observation period using the Wilcoxon signed rank test for the fourth week of the treatment period.

4. Degree of straining: patient diary

In the fourth week of the treatment period, the frequency and rate of the degree of straining will be calculated and compared with the second week of the observation period by the Wilcoxon signed rank test.

5. Presence of a sense of incomplete evacuation: patient diary

The frequency and percentage of the presence or absence of a sense of incomplete evacuation at each week of the treatment period will be calculated and compared with the second week of the observation period by the McNemar test for the fourth week of the treatment period.

6. Satisfaction with treatment: patient questionnaire

Summary statistics will be calculated for treatment satisfaction at each week of the treatment period, and the results will be compared with those of the second week of the observation period using the Wilcoxon signed rank test for the fourth week of the treatment period.

7. Spontaneous bowel movement (SBM) frequency

Summary statistics will be obtained for the number of SBMs during the 7-day period (total number of SBMs during the 7-day period) at each week of the treatment period, and the corresponding t-test will be performed for the change in the number of SBMs during Week 4 of the treatment period from Week 2 of the observation period.

8. Complete spontaneous bowel movement (CSBM) frequency

Summary statistics will be obtained for the number of CSBMs during the 7-day period (total number of CSBMs during the 7-day period), and corresponding t-tests will be performed for the change in the number of CSBMs during Week 4 of the treatment period from Week 2 of the observation period.

9. Fecal hardness based on the Bristol stool form scale

Summary statistics of fecal hardness at each week of the treatment period will be calculated and compared with the second week of the observation period by the Wilcoxon signed rank test for the fourth week of the treatment period.

(2) Comparison of the fourth week of the treatment period with the second week of the observation period

1. Constipation Score (CSS)

Summary statistics will be calculated for the total constipation score and compared using the Wilcoxon signed rank test. Summary statistics are also calculated for each symptom and compared by Wilcoxon signed rank test.

2. JPAC-QOL score

Corresponding t-test for change in JPAC-QOL score.

3. Comparison of absolute values and percent composition of bile acid concentrations in feces

Corresponding t-test is performed for the change in bile acid concentration.

(3) Time from taking elobixibat to defecation in each week of the treatment period.

Summary statistics were calculated for the time from elobixibat administration to the first bowel movement in 7 days (median of 7 days) at each week of the treatment period, and the trends were examined.

(4) Examining the relationship between the evaluation items

To examine the relationship between the endpoints such as improvement of loss of defecation desire, patient satisfaction, and bile acid concentration.

16.3.3 Safety Analysis

The number and percentage of cases of SAS will be calculated by disease event and severity.

Vital signs and clinical laboratory values are summarized at each observation time point, and changes over time are shown in graphs.

16.4 Intermediate analysis

No interim analysis will be performed.

16.5 Procedures for handling missing, rejected and abnormal data

In principle, missing values will not be supplemented in the analysis. However, in the case of discontinued patients, if the study drug has been taken for more than 1 week according to the definition in 9.1, missing values will be supplemented by evaluation at the time of discontinuation. Rejected and abnormal data will be reviewed by the Principal Investigator/Research Office and the Data Center prior to data fixation.

16.6 Procedure for changing the statistical analysis plan

If there are any changes from the original statistical analysis plan in this study, the research protocol will be revised and explained in the summary report of this study.

**17. quality control and quality assurance**

17.1 Access to source documents and source materials, etc.

The source documents in this study are as follows.

Record of subject's consent

　　Medical records

　　Inspection records

　　Records directly written by the subject (patient questionnaire, patient diary, questionnaire, etc.)

　　Documents or records pertaining to the research

　　Other

In this study, the principal investigator and the implementing medical institution will provide direct access to all clinical research-related records, including source documents, during monitoring, auditing, and investigations by accredited clinical research review committees and regulatory authorities related to the clinical research in question.

17.2. data management

In this study, data management will be performed by the data center staff. The data entered in the electronic case report form, such as outliers and abnormal values confirmed by data monitoring, will be checked against electronic case report forms as queries and requests for correction will be made. After the data is fixed at the data center, the fixed data will be provided to the person responsible for statistical analysis. Details will be specified in the data management plan.

## 17.3. central monitoring

The data center shall cover the contents entered into the EDC system and refer to the results of the processing of the electronic data. The data center prepares a central monitoring report about once a year (at an unspecified time) in response to a request from the principal investigator. The person in charge of the data center submits the central monitoring report compiled by the central monitoring to the principal investigator and the research secretariat. The principal investigator shall share the problems pointed out in the central monitoring report with the principal investigator and make efforts to improve them.

Central monitoring items

1. Registration status: number of registrations - cumulative/period

2. Eligibility: ineligible cases/potentially ineligible patients

3. Pre-treatment background factors

4. Protocol treatment in progress/completed, reason for discontinuation/termination

5. Non-compliance/Disease etc.

6. Serious non-compliance

7. Serious diseases, etc.

8. Adverse reactions/adverse events

9. Other issues related to study progress and safety

10. Problems with data cleaning

## 17.4. audit

No audit will be conducted in this study.

# **18. ethical matters**

## 18.1 Regulations to be observed

This study is financially supported by Mochida Pharmaceutical Co., Ltd. and EA Pharma, Inc. and therefore falls under the category of specified clinical research under the Clinical Research Act (Act No. 16 of 2017). Therefore, this study will be conducted in compliance with the Clinical Research Act and in accordance with the ethical principles of the Declaration of Helsinki.

## 18.2 Handling of Personal Information, etc.

Information that may identify the subject, such as the subject's name, will not be made known to outside parties by the implementing medical institution. Subjects are identified and referred to using the case registration number, subject identification code, and gender issued at the time of provisional registration, so that a third party cannot identify the subject by name or other information. Each medical institution should appropriately manage the case registration number, etc. using a corresponding table. When a subject's case report, test data, specimens, etc. are provided outside of the relevant medical institution, they must be processed or managed so that the subject cannot be immediately identified.

## 18.3 Expected Benefits and Disadvantages to Subjects from Participation in Research

18.3.1. Expected benefits

Subjects are not expected to accrue any direct benefit from participation in this study.

Research participation may contribute to future medical advances.

18.3.2. Foreseeable disadvantages

During the study period, examinations, medical examinations, and medication administered in this study will be performed within the scope of normal medical care, and subjects will be required to pay a portion of their own costs according to health insurance coverage.

The research drug used in this study is approved for indication and covered by insurance for the target diseases of this study. Diseases, etc. that may occur in the subject are judged by those described in "12.4. Predictability". If Diseases, etc. occur, the investigator or the investigator will take appropriate measures in accordance with "12.5. Measures to be taken when Diseases, etc. occur". There is a possibility that the number of visits and examinations will increase due to treatment for diseases, etc.

## 18.4 Handling of research results (including incidental findings) pertaining to subjects

Although there is no possibility that this research will yield important findings regarding the health of subjects, genetic characteristics that can be passed on to offspring, etc., if information (including incidental findings) that may have a significant impact on the health of subjects is obtained through tests, etc., the principal investigator or sub-investigator will explain the information to the subjects and take appropriate measures, such as treatment and Appropriate measures will be taken. In addition, the results of individual subjects' participation in the study will be explained to the subjects themselves in the course of medical treatment.

# **19. Cost burden and compensation for subjects**

During the study period, examinations, medical examinations, and medication administered in this study will be performed within the scope of normal medical care, and subjects will be required to pay a portion of their own costs according to the coverage of their health insurance.

In this study, subjects will be paid a burden reduction fee of 5,000 yen, paid through prepaid cards (maximum 15,000 yen) for each visit. The burden reduction fee will be provided from the research funds provided by Mochida and EA Pharma.

# **20. Compensation for health damage**

In the event of health damage to subjects, appropriate treatment will be provided within the scope of insurance. Subjects will be responsible for their own medical expenses. We will purchase clinical research liability insurance to provide compensation in the event of liability for health damage resulting from this research, or in the event of death or disability level 1 or 2 to the subject. Compensation is subject to certain conditions, which may exclude or limit the payment of compensation. If the subject is negligent, it will not be covered. If the physician is at fault, compensation will be provided by the physician liability insurance.

# **21. Funding sources and conflicts of interest**

## 21.1. funding sources and financial relationships

This study will be funded by Mochida and EA Pharma. In order to receive funding, an investigator-initiated clinical research agreement will be concluded. Mochida Pharmaceutical Co., Ltd. and EA Pharma Co., Ltd. will prepare a research protocol in consultation with the principal investigator, but will not be involved in decision-making regarding the conduct, analysis, or publication of this study.

## 21.2 Management of conflicts of interest

21.2.1 Management of conflicts of interest by principal investigators or principal investigators

The principal investigator shall establish conflict of interest management standards, conduct fact-finding by the administrator of the implementing medical institution, and prepare a conflict of interest management plan. The Principal Investigator shall listen to the opinions of the Accredited Clinical Research Review Committee described in the protocol regarding the conflict of interest management standards and the conflict of interest management plan, and shall manage them appropriately.

21.2.2. Management of Conflicts of Interest of Principal Investigators, etc.

In this research, the principal investigator, subordinate investigators, and the chief statistician, as those who report conflicts of interest, shall confirm the facts with the administrator of the medical institution in which the research is conducted, and obtain a conflict-of-interest confirmation report. The Principal Investigator shall prepare a Conflict-of-Interest Management Plan based on the contents of such Conflict of Interest Confirmation Report, and shall listen to the opinions of the Accredited Clinical Research Review Committee described in the Implementation Plan, and shall manage the conflict appropriately.

# **22. Compliance with the research protocol, management of changes and non-compliance (e.g., deviations from the research protocol)**

## 22.1 Compliance with research protocols

The principal investigator or sub-investigator must not do anything that does not conform to the research protocol (i.e., deviate from or change the research protocol) without the prior agreement of the principal investigator and the prior written approval based on the review of an accredited clinical research review committee described in the protocol.

## 22.2 Changes to the research protocol

If any changes are made, the principal investigator submits the revised implementation plan and a notification of minor changes in the implementation plan or a notification of changes in the implementation plan to an accredited clinical research review committee for approval. After approval, the research is registered in jRCT and the implementation plan is submitted to the Minister of Health, Labor and Welfare after approval is obtained from the administrator of the medical institution. If the submission is accepted and published on jRCT, the accredited clinical research review committee is notified and the administrator of the implementing medical institution is informed. In addition, information is provided to each principal investigator, and the principal investigator reports to the administrator of the medical institution.

## 22.3 Management of non-compliance (e.g., deviation from the research protocol)

The sub-investigator who becomes aware of the noncompliance shall report it to the principal investigator. The principal investigator shall promptly report to the administrator of the medical institution and further notify the principal investigator. The principal investigator shall provide information to all principal investigators. The Principal Investigator and each Principal Investigator shall also report to the administrator of the medical institution concerned. The Principal Investigator and the Principal Investigator of the institution where the non-compliance occurred shall appropriately consider measures to prevent recurrence.

If the principal investigator judges that the non-compliance that has occurred is a particularly serious non-compliance, the principal investigator shall promptly prepare a Uniform Form 7 Serious Non-compliance Report and submit it to the Accredited Clinical Research Review Committee for its opinion.

If there is a concern that the principal investigator may not report the non-compliance to the appropriate party, the subcontracting physician may make the report directly.

Non-compliance that do not comply with the research protocol for the purpose of avoiding immediate danger to subjects or for other unavoidable medical reasons are not included in serious non-compliance. Therefore, if the non-compliance is unavoidable, it is not necessary to report it to the accredited clinical research review committee, but even in that case, a record of the non-compliance should be made.

# **23. Periodic report**

## 23.1 Periodic reporting to the Accredited Clinical Research Review Committee

(2) The principal investigator shall report the implementation status of the specified clinical research to the administrator of the host medical institution every year (within 2 months after the expiration of the relevant period) from the date of submission of the implementation plan to the Minister of Health, Labour and Welfare, and shall make a periodic report to the accredited clinical research review committee described in the implementation plan. The items to be reported on the implementation status shall be as follows

- - 1. Number of subjects who participated.
    2. (Occurrence and Subsequent Course of Diseases, etc.)
    3. Occurrence of non-compliance and subsequent actions
    4. Evaluation of safety and scientific validity
    5. (Matters concerning involvement of a distributor of pharmaceuticals, etc. specified in the conflict of interest management standards)

(2) When the Principal Investigator has made a report to the Accredited Clinical Research Review Committee, he/she shall promptly provide information to the other Principal Investigators to that effect. The other principal investigators shall promptly report the contents of such information provision to the administrator of the other medical institution.

## 23.2 Periodic reporting to the Minister of Health, Labour and Welfare

The principal investigator shall report to the Minister of Health, Labour and Welfare on the following matters within one month from the date on which the accredited clinical research review committee described in the research plan gives its opinion on the implementation status of the specified clinical research.

1. Name of the committee as stated in the implementation plan
2. (2) The appropriateness of the continuation of the said specific clinical research by the Committee
3. Number of subjects who participated in the specified clinical research

# **24. Disclosure of information on research and publication of results**

## 24.1 Registration of Studies

Prior to the implementation of this study, the data will be recorded (registered) in a clinical trial database maintained by the Ministry of Health, Labor and Welfare (jRCT). The database will be updated as appropriate according to changes in the research protocol and the progress of the study. When the study is completed, the results of the study will be registered.

## 24.2 Publication of research results

The principal investigator shall prepare the primary or summary endpoint report and its summary. The deadline for the preparation of the report shall be within one year after the end of the period for the collection of data pertaining to the primary endpoint or all endpoints.

(2) When the principal investigator prepares the primary endpoint report or the summary report and its outline, the principal investigator shall obtain the opinion of the accredited clinical research review committee described in the protocol, submit it to the administrator of the research institution without delay, and record (register) the summary of the primary endpoint report or the summary report in jRCT. The term "without delay" means within one month from the date on which the committee expressed its opinion. At the time of submission to the administrator of the medical institution, the principal investigator shall provide information to the other principal investigators, and the other principal investigators shall promptly report the contents of the information provided to the administrator of the other medical institution.

## 24.3 Publicizing of results at conferences, etc.

The results obtained from this research will be promptly publicized by presenting them at a conference or submitting a paper. When the results are published, necessary measures will be taken to protect the human rights of the subjects and their related persons, or the rights and interests of the researchers and their related persons. The presenter of the conference presentation and the first author of the paper will be determined through consultation.

# **Attribution of research results (intellectual property rights)**

If any inventions, etc. arise as a result of the conduct of this research, the handling of intellectual property rights based on such inventions, etc. shall be discussed with Mochida Pharmaceutical Co. However, the rights related to the Study drug shall belong to EA Pharma Co.

# **26. System for conducting research**

See Attachment 1

# **27. participating institutions and institutional principal investigators**

See Attachment 2

# **28. References**

1] Acosta A, Camilleri M. Elobixibat and its potential role in chronic idiopathic constipation.

Ther Adv Gastroenterol. 2014; 7(4): 167-75

[2]Bampton PA., Dinning PG., Cook IJ. et al. The proximal colonic motor response to rectal mechanical and chemical stimulation. Am. J. Physiol. Gastrointest. Liver Physiol. 2002:282:G443.

[3]Edwards CA., Brown S., Read NW. et al. Effect of bile acid on anorectal function in man. Gut 1989:30:3.

[4]Ohkubo H., Takatsu T., Yoshihara T. et al. Difference in defecation desire between patients with and without chronic constipation: A large-scale internet survey. Clin Transl Gastroenterol. 2020:11:e00230.

[5] Nakajima A, Seki M et al. Safety and efficacy of elobixibat for chronic constipation: results from a randomised, double-blind, placebo-controlled, phase 3 trial and an open-label, single-arm, phase 3 trial. Lancet Gastroenterol Hepatol. 2018 Aug;3(8):537-547,

[6] Guidelines for the Treatment of Chronic Constipation 2017

[7] Shawn X. Sun ., Marco DiBonaventura., Impact of Chronic Constipation on Health-Related Quality of Life, Work Productivity, and Healthcare Resource Use: An Analysis of the National Health and Wellness Survey: Dig Dis Sci (2011) 56:2688–2695

[8]J.F.Johanson., J.Kralstein., Belsey., Chronic constipation: a survey of the patient perspective: Aliment Pharmacol Ther 25, 599–608 Journal compilation 2007 Blackwell Publishing Ltd

[9] Japanese Geriatrics Society: Guidelines for safe drug therapy for the elderly 2015；108-120

[10] Nakashima, Jun. Basic strategy of internal medicine therapy What is the treatment strategy with high patient satisfaction? Essential Knowledge of Chronic Constipation Management for Clinicians Iyaku Journal 2015；130-40

[11] Toshihiko Tomita, Tadayuki Oshima, Hiroto Miwa. Stimulant Laxatives - Anthraquinone and Diphenol Derivatives. Medicina 2016； 53： 1374-7

[12] The Japanese Gastroenterological Association: Guidelines for the treatment of functional gastrointestinal disorders 2014 - Irritable bowel syndrome (IBS) Nanjiang-do 2014

[13] Partial Amendments to the Notes on Changes in Indications and Effects under the Pharmaceutical and Medical Devices Law" (Hōshihatsu No. 0821, No. 1, August 21, 2008)

[14] Partial Revision of the National Health Insurance Drug Price (NHI) Standards for Drugs in Use" (Hōhi-ihatsu 1127, No. 2, November 27, 2008)

[15] Guideline on the evaluation of medicinal products for the treatment of chronic constipation (including opioid induced constipation) and for bowel cleansing. European Medicines Agency

# **29. Appendix**

A：Patient Questionnaire

B：Constipation severity score（CSS）

C：Bristol stool form scale (BS score)

D：PAC-QOL　Japanese version

E：Diagnostic Criteria for Functional Constipation in Rome IV
